# PiRNA-4447944 promotes castration-resistant growth and metastasis of prostate cancer by inhibiting NEFH expression through forming the piRNA-4447944-PIWIL2-NEFH complex

**DOI:** 10.7150/ijbs.96173

**Published:** 2024-07-01

**Authors:** Qiang Peng, Yu Chen, Tingting Xie, Dandan Pu, Vincy Wing-Sze Ho, Jingkai Sun, Kang Liu, Ronald Cheong-Kin Chan, Xiaofan Ding, Jeremy Yuen-Chun Teoh, Xin Wang, Peter Ka-Fung Chiu, Chi-Fai Ng

**Affiliations:** 1 SH Ho Urology Centre, Department of Surgery, The Chinese University of Hong Kong, Hong Kong, China.; 2 State Key Laboratory of Biotherapy and Cancer Center, West China Hospital, Sichuan University and Collaborative Innovation Center for Biotherapy, Chengdu, Sichuan, China.; 3 Department of Biomedical Sciences and Tung Biomedical Sciences Centre, City University of Hong Kong, Hong Kong, China.; 4 HitGen Inc., Building 6, No.8 Huigu First East Road, Tianfu International Bio-Town, Shuangliu District, Chengdu, Sichuan, China.; 5 Department of Surgery, Sir Y.K. Pao Centre for Cancer, The Chinese University of Hong Kong, Hong Kong, China.; 6 Department of Anatomical and Cellular Pathology, The Chinese University of Hong Kong, Hong Kong, China.; 7 Faculty of Health Sciences, University of Macau, Macau, China.; 8 Department of Surgery, The Chinese University of Hong Kong, Hong Kong, China.

**Keywords:** PIWI-interacting RNAs, prostate cancer, castration resistance, NEFH, piR-4447944

## Abstract

Castration-resistant prostate cancer (CRPC) is the leading cause of prostate cancer (PCa)-related death in males, which occurs after the failure of androgen deprivation therapy (ADT). PIWI-interacting RNAs (piRNAs) are crucial regulators in many human cancers, but their expression patterns and roles in CRPC remain unknown. In this study, we performed small RNA sequencing to explore CRPC-associated piRNAs using 10 benign prostate tissues, and 9 paired hormone-sensitive PCa (HSPCa) and CRPC tissues from the same patients. PiRNA-4447944 (piR-4447944) was discovered to be highly expressed in CRPC group compared with HSPCa and benign groups. Functional analyses revealed that piR-4447944 overexpression endowed PCa cells with castration resistance ability *in vitro* and *in vivo*, whereas knockdown of piR-4447944 using anti-sense RNA suppressed the proliferation, migration and invasion of CRPC cells. Additionally, enforced piR-4447944 expression promoted *in vitro* migration and invasion of PCa cells, and reduced cell apoptosis. Mechanistically, piR-4447944 bound to PIWIL2 to form a piR-4447944/PIWIL2 complex and inhibited tumor suppressor NEFH through direct interaction at the post-transcriptional level. Collectively, our study indicates that piR-4447944 is essential for prostate tumor-propagating cells and mediates androgen-independent growth of PCa, which extends current understanding of piRNAs in cancer biology and provides a potential approach for CRPC treatment.

## Introduction

Prostate cancer (PCa) is one of the most common male urinary malignancies, with the highest morbidity, and is the fifth leading cause of cancer-related mortality in men worldwide 1. Androgen deprivation therapy (ADT) is the first-line option for patients diagnosed with advanced/metastatic PCa 2. Despite initially responding to ADT at the hormone-sensitive stage, all patients will inevitably become unresponsive and progress to a more aggressive and deadly form of the disease, namely castration-resistant prostate cancer (CRPC) 3. Currently, CRPC is an incurable disease, and delineating the molecular mechanisms that contribute to PCa cells growth under androgen-deprived conditions is essential for discovering novel therapeutic strategies to delay or reverse the progression of the disease. Accumulating evidence shows that during CRPC progression, PCa cells use a variety of androgen receptor (AR)-dependent or AR-independent pathways to survive in the androgen-deprived environment 4. However, there is still a lack of novel molecular targets involved in the progression of castration resistance, which poses a big challenge to the treatment of CRPC. Thus, further research on CRPC is an essential step towards developing therapies against this lethal phenotype.

Various studies have revealed that non-coding RNAs (ncRNAs) play essential roles in tumorigenesis, progression, and prognosis of human cancers, providing a strong rationale for developing ncRNAs-based therapeutics 5, 6. PIWI-interacting RNAs (piRNAs) are a class of small ncRNAs discovered in germ and somatic cells that specifically interact with PIWI proteins 7. While initial investigations into piRNAs in flies and mice focused on their presence in germline cells, subsequent research has revealed that piRNAs are also present and active in somatic cells 8, 9. Recent studies have shown that piRNAs are expressed in a tissue-specific manner in various human tissues and modulate key signaling pathways at the transcriptional or post-transcriptional level in the physiological and pathological processes 10, 11. At the level of post-transcriptional gene regulation, cytoplasmic piRNAs can impede target function by directly binding to a wide range of downstream target genes, resulting in RNA repression/degradation due to the base-pairing between piRNAs and target RNAs 12-14. Such regulation was presented, Peng et al. found that piR-55490 bound to the 3′UTR mRNA of mTOR, leading to the degradation of mTOR and consequent suppression of lung cancer development 15. Similarly, piR-30188 was found to suppress the malignant phenotype of glioma cells by repressing the expression of long ncRNA OIP5-AS1 through sequence complementarity 16. The differentially expressed piRNAs in tumor and normal cells indicate their cancer-specific roles, and increasing evidence showed that piRNAs could affect the expression of both oncogenes and tumor suppressor genes, thereby contributing to cancer development and progression 17. In contrast to the growing body of studies that underpin the miRNA-cancer connection, the roles of piRNAs in PCa progression, particularly in CRPC, remain currently in its infancy.

To address this, we performed a discovery step using small RNA sequencing-based expressing profiling to identify CRPC-related piRNAs. We then identified a CRPC-specific piRNA, piRNA-4447944 (piR-4447944), by validating candidate piRNAs in other patient cohorts and cell lines. Subsequently, we investigated the functional roles and underlying mechanisms of piR-4447944 in PCa cells. Our findings suggest that piR-4447944 promotes PCa castration-resistant growth and is a multi-functional and promising therapeutic target.

## Materials and Methods

### Patient samples

A total of 10 fresh benign prostate tissues, 20 hormone-sensitive PCa (HSPCa) FFPE tissues and 20 CRPC FFPE tissues were obtained from the Prince of Wales Hospital, the Chinese University of Hong Kong. Informed consent was obtained from all patients, and ethical approval was obtained from the Joint Chinese University of Hong Kong-New Territories East Cluster Clinical Research Ethics Committee. Benign prostate tissues and HSPCa tissues were collected at the time of biopsy, and CRPC tissues were collected at the time of transurethral resection of the prostate (TURP) surgery when the patients progressed to CRPC stage. Among these 20 HSPCa tissues and 20 CRPC tissues, 9 pairs of tumor tissues (HSPCa and its corresponding CRPC) from the same patient were included for small RNA sequencing. Next, the expression levels of candidate piRNAs from sequencing results were validated in a bigger cohort. Case series details such as age, pathological stage, PSA value and Gleason score are shown in Table S1.

### RNA isolation and RT-qPCR assays

Tumor tissue sampling areas were identified by pathologists, and 8 sections of 5 μm in the thickness of each formalin-fixed paraffin-embedded (FFPE) tissue were taken for RNA extraction. Total RNA, including small RNAs, from FFPE tissues was extracted with miRNeasy FFPE Kit (Qiagen, # 217504) following the manufacturer's protocols. The total RNA of fresh benign tissues or cells was extracted using miRNeasy Mini Kit (Qiagen, #217004) according to the manufacturer's instructions. The quality and concentration of the RNA were measured by NanoDrop 2000 Spectrophotometers and kept at -80 °C.

For piRNA expression, total RNA was reverse transcribed using piRNA specific primer with TaqMan microRNA Reverse Transcription Kit (Applied Biosystems, #4366596). The expression of piRNAs was analyzed using Custom TaqMan small RNA assays (Applied Biosystems, #4398987) on the Applied Biosystems QuantStudio 7 Flex Real-Time PCR system, according to the manufacturer's protocols. The primer sequences used are shown in Table S2. To quantify piRNAs, U6 small nuclear RNA was employed as an internal control, and the average expression levels of tissue piRNAs were normalized against U6 using the 2^-ΔCt^ method.

For mRNAs, 1 ug of total RNA was reverse-transcribed to cDNA using SuperScript™ III First-Strand Synthesis System kit (Invitrogen, #18080051). Real-time PCR was thereafter performed using PowerUp™ SYBR™ Green Master Mix (Applied Biosystems, #A25779) according to the instructions. The reaction mixture was conducted under the Applied Biosystem QuantStudio 7 Flex system (California, USA). GAPDH was used as a normalizer for mRNA. The relative quantification values were calculated by 2^-ΔΔCt^ method. The primer sequences used are shown in Table S3.

### Small RNA sequencing and data analysis

To identify CRPC-associated piRNAs, we performed small RNA sequencing on 10 benign prostate tissues, 9 HSPCa tissues and their paired CRPC tissues. Briefly, 1μg of total RNA was used for library preparation with BGI UMI library preparation kits and protocols. The general process for constructing the small RNA library is as follows: small RNA 18-40 nt in length was isolated on a 15% polyacrylamide gel. Then the 18-40 nt small RNAs were ligated to adenylated 3' adapters annealed to unique molecular identifiers (UMI), followed by the ligation of 5'adapters. Purified RNAs were reverse-transcribed to cDNA with UMI-labeled primer and the library was constructed and validated. Sequencing was performed with the BGISEQ-500 platform (BGI, Hong Kong, China) with the single-end (SE) mode.

For data analysis, we used fastqc (version 0.11.9, https://github.com/s-andrews/FastQC) and cutadapt (version 2.6) to quality control and moved adapters and low-quality bases in reads. Database piRNABank (http://pirnabank.ibab.ac.in/) and piRBase (http://bigdata.ibp.ac.cn/piRBase/) were used for annotation. The wholemapping ratio of different small RNAs in our samples was shown in Table S4. Then we apply Bowtie (https://sourceforge.net/projects/bowtiebio/files/bowtie/1.3.1/), to Genome Reference Consortium Human GRCh38 (hg38, https://hgdownload.soe.ucsc.edu/) from UCSC and extracted the aligned reads for further analysis. FeatureCounts (version 2.0.3, http://subread.sourceforge.net/) was utilized to calculate the coverage of piRNAs. The quantified small RNA sequencing data was analyzed to identify differentially expressed piRNAs from different groups. The differential expression analysis was conducted using limma package (version 3.44.3).

### Construction of castration-resistance LNCaP-AI prostate cancer cell line

We established an androgen-independent LNCaP-AI cell line derived from hormone-sensitive LNCaP cell line to mimic the traits of castration resistance *in vitro*. LNCaP cells were exposed to androgen deprivation conditions for six months by gradually reducing androgen level in the medium every week, according to previously reported protocol 18, 19. Briefly, LNCaP-AI cells were developed by the prolonged culture of LNCaP cells in RPMI-1640 medium supplemented with 10% charcoal-stripped Fetal Bovine Serum (CS-FBS) (Gibco, #12676029). To validate the establishment of LNCaP-AI, a PSA-ELISA kit (Invitrogen, #EHKLK3T) was used to detect total PSA secretion level by collecting medium supernatants at the indicated time points according to the protocol. The protein expression levels of cells were detected by western blot, and cell proliferation ability was measured using a CCK-8 assay.

### Cell lines, RNA oligos, antisense and transfection

Human PCa cell lines, 22Rv1, VCaP, PC3 and DU145 were obtained from ATCC (Manassas, USA). PCa cells LNCaP and normal immortal prostate epithelial cell line RWPE-1 were kindly supplied by Prof. CHAN Leung Franky, The Chinese University of Hong Kong. RWPE-1 was maintained in a defined Keratinocyte serum free medium with bovine pituitary extract (BPE) and human recombinant epidermal growth factor (EGF). LNCaP and 22Rv1 were cultured in RPMI-1640 medium, and VCaP was maintained in DMEM medium. DU145 cells were grown in Eagle's minimum essential medium, and PC3 cells were grown in F-12K medium (Gibco). All cell media were supplemented with 10% fetal bovine serum (FBS; Gibco). LNCaP-AI cells were cultivated in RPMI-1640 medium supplemented with 10% CS-FBS (Gibco). Cells were incubated at 37 ℃ with 5% CO2.

piRNA mimics, piRNA inhibitors and negative controls were designed and purchased from Ribobio (Guangzhou, China). For the overexpression of piRNA, piRNA mimic was designed using its mature sequence and synthesized as a monophosphate and 2′ O-methoxy modified RNA oligo. For the inhibition of piRNA in PCa cells, we designed antisense oligos as described in Table S5. piRNA mimic/inhibitor and their corresponding negative controls (NC-mimic/NC-inhibitor) were transfected into PCa cells using lipofectamine-3000 following the manufacturer's instructions. Then, the cells were incubated for 6 h, after which the transfection mixture was replaced by a complete medium and incubated for desired periods required for subsequent molecular functional assays. The transfection efficiency was verified by RT-qPCR.

### Overexpression of neurofilament heavy polypeptide (NEFH)

The plasmid pcDNA3.1 and pcDNA3.1-NEFH were purchased from Addgene (Massachusetts, USA). The NEFH-pCMV-SPORT6 clone was digested serially with EcoR1 and Xba1, and then was constructed into pcDNA3.1 after digestion of pcDNA3.1 with the same enzymes. The plasmid pcDNA3.1-NEFH and corresponding control-vectors were transfected with lipofectamine 3000 according to the manufacturer's protocol. The overexpression of NEFH in PCa cells was confirmed by western blot.

### Western blot

Total proteins were extracted using M-PER™ Mammalian Protein Extraction Reagent (Thermo Scientific, #78501) with a proteinase inhibitor (Thermo Scientific, #78420) and quantified protein concentration by Bradford protein assay (Bio-Rad, #5000006). Before electrophoresis, the protein was denatured with the 1X protein loading dye at 95 ℃ for 10 min. The protein samples were subjected to separation through either 10% or 12% SDS-PAGE gels and then transferred onto 0.2 μm PVDF membranes (Bio-Rad, #1620177). Subsequently, the membranes were blocked at a temperature of 37 °C for 1 h, utilizing 5% skim milk. This was succeeded by an overnight incubation at 4 °C with antibodies (Table S6). A secondary antibody (CST, USA) and ECL chemiluminescence reagents (Bio-Rad, #1705062) were used to visualize the target proteins.

### Cell proliferation assay

The cell counting kit 8 assay (CCK8, #ab228554) was used to detect cell viability, and a standard protocol from Abcam was followed. Briefly, PCa cells (4,000/well) were seeded into 96-well plates and cultured in 200 μl normal medium with FBS or CS-FBS medium. Then the absorbance was measured at a wavelength of 450 nm at the indicated times using an automatic microplate reader (µQuan).

For the colony formation assay, PCa cells were seeded in a 96-well plate at a density of 500 cells per well. After two weeks in the medium with CS-FBS, the clones were washed with 1× phosphate buffered saline (PBS) and stained with crystal violet for approximately 20 min. The clones were then imaged and quantified.

An EdU staining proliferation kit was used for imaging (Invitrogen, #C10639). PCa cells (2×10^5^/well) were seeded in a 12-well plate and cultured in medium with CS-FBS for 48 h, and another 6 h incubation with 10 μmol/L 5-ethynyl-2′-deoxyuridine (EdU), then fixation, permeabilization, and EdU staining, which were performed according to the manufacturer's instructions. After the process, the cells were stained with DAPI (Invitrogen), and viewed under a fluorescence microscopy (Leica, German).

### Wound-healing and transwell assays

For the wound-healing assay, PCa cells in the 6-well plate were starved with serum-free medium overnight and scratched with sterilized tips. The wound closure was measured at 0 h and 72 h, and the plates were photographed.

Transwell assays were performed in the Boyden chambers (Corning) using 8μm pore size membrane coated with Matrigel (invasion assay) or without Matrigel (migration assay). PCa cells in serum-free medium were added into the upper chamber, and 600 μl of cell growth medium containing 10% FBS was supplied in the lower chamber. After 72 h incubation, PCa cells of the filters were fixed with methanol for 15 min, stained with 0.1% crystal violet for 20 min and counted in an inverted microscope (Olympus, Japan).

### Flow cytometric analysis

For cell cycle analysis, cells were harvested and washed twice with PBS, and then fixed with 70% ice-cold ethanol overnight at 4 °C. Next day, after washing cells with PBS and staining buffer, the single-cell suspensions were stained with 500 ul PI/RNase Staining Buffer (BD Biosciences, #550825). Cells were analyzed by Beckman Cytoflex LX Flow Cytometer, and the cell cycle distributions were analyzed in FlowJo v10.8.1 (FlowJo, USA).

For apoptosis analysis, cells were cultured with 10% CS-FBS and the corresponding IC_50_ of enzalutamide (MedChemExpress, #HY-70002) for 48 h. The FITC-Annexin V Apoptosis Detection Kit I (BD Biosciences, #556547) was used to quantify apoptotic cells following the provided protocols. In brief, cells were harvested and stained with Annexin V-FITC and propidium iodide (PI) to label apoptotic cells. The percentage of apoptotic cells was measured by flow cytometry system (Beckman Cytoflex LX). Detailed information on the antibodies used for flow cytometry is included in Table S6.

### Fluorescence *in situ* Hybridization (FISH) assay

The FISH kit (GenePharma, China) was used to localize piR-4447944 in PCa cells. The cells were seeded in a 24-well plate at a density of 5×10^4^ cells/well and cultured overnight in an incubator. After washing with PBS, cells were fixed in 4% paraformaldehyde and then permeabilized in PBS containing 0.5% Triton X-100, and then pretreated with pre-hybridization buffer at 37 ℃ for 30 min. Subsequently, cells were hybridized with 5 μM Cy3-labeled RNA of piR-4447944 FISH probe mix in a moist chamber at 42 °C 12 h. After hybridization, cells were stained with DAPI for 10 min at room temperature, followed by observation under a fluorescent microscope. Cy3-labeled piR-4447944 fluorescent probe sequence (5'-3') was “Biotin-CCAACATTTTCTGGGTATGGGCCCGATAGCTTA".

### Nuclear-cytoplasm separation assay

1×10^6^ PCa cells were washed twice with PBS. We performed nuclear-cytoplasm separation experiment using PARIS kit (Invitrogen, USA) according to the manufacturer's instructions. The expression levels of piRNAs as well as controls (GAPDH and U6) in the nuclear and cytoplasm were detected by RT-qPCR.

### Dual-luciferase reporter assay

The wild-type (WT) and mutant (MUT) 3′-UTR sequences of NEFH mRNA (WT-NEFH, MUT-NEFH) sequences were cloned into the pmiR-RB-Report^TM^ dual-luciferase vector to generate NEFH WT/MUT plasmids (Ribobio, China). Cells were co-transfected with 100 nM piR-4447944 mimic or NC-mimic and 100 ng of a dual luciferase vector expressing the WT-NEFH or MUT-NEFH using lipofectamine-3000 reagent. After 48 h, the luciferase activities were measured using the dual-luciferase reporter assay system (Promega, USA) according to the manufacturer's protocol. Renilla luciferase activity was normalized to firefly luciferase activity.

### RNA immunoprecipitation (RIP) assay

RNA immunoprecipitation (RIP) assay was performed with the Magna RIP kit (Merck Millipore) based on the guidelines. The purpose of RIP was to extract and identify the RNAs bound to PIWI proteins. 1 × 10^7^ cells were lysed with RIP lysis buffer with one freeze-thaw cycle. After incubation with anti-PIWIL2, anti-PIWIL4 and IgG antibody for 30 min, the magnetic beads were mixed with 100 μl of cell lysate supernatant respectively, rotated and incubated overnight at 4 °C. The beads were then harvested and washed thoroughly. Next, RNA was extracted and RT-qPCR was performed. The amplification product of piR-4447944 was detected by electrophoresis on a 3% agarose gel. IgG was used for negative control. Primer used for NEFH quantification was designed as follows: forward: 5'-GGGGCCTCCTTCTTCAAACA-3', reverse: 5'-TGTTTACGTGTGGCATTCGG-3'.

### RNA pull-down

The RNA Pulldown assay was performed using a miRNA pull-down kit (BersinBio, China). Briefly, PCa cells in 10 cm dishes were transfected with biotin-labeled piR-4447944 mimic or negative control mimic (GenePharma, 100 nM), and lysed after 48 h incubation. Simultaneously, streptavidin magnetic beads were mixed with cell lysates and incubated with mild rotation (20 rpm/min) for 4 h at 4 °C to pulldown the biotin-coupled RNA complex. Beads were then washed to remove unbound materials. The RNA was eluted, isolated, and subjected to RT-qPCR analysis.

### High-throughput mRNA sequencing

To investigate the regulatory role of piR-4447944 on genome-wide target mRNAs, we treated PCa cells 22RV1 and LNCaP with NC or piR-4447944 mimic, and then extracted total RNA for mRNA sequencing and bioinformatic analysis. The sequencing reads were aligned to the human genome (hg38) using STAR 2.7.1a 20 and gene expression was quantified by RSEM v1.3.0 21. Differentially expressed genes (DEGs) were determined with R 4.1.1 using the package DESeq2 1.34.0 22. The log2 fold change (L2FC) values were shrunk with the DESeq2 function lfcShrink (type="normal") to control for variance of L2FC estimates for genes with low read counts. Genes with p value < 0.05 and log2|FC| > 0.2 were considered as differentially expressed.

### piR-4447944 target prediction

The sequence of piR-4447944 was retrieved from the piRBase. Based upon piRNA:mRNA sequence complementarity, we used miRanda program (version 3.3a, http://cbio.mskcc.org/microrna_data/miRanda) to search for targets of piR-4447944 against the transcripts of those down-regulated genes. The mRNA sequence of down-regulated genes was retrieved from NCBI. The candidate mRNAs were selected based on alignment score ≥140 and binding energy ≤ -20.0 kcal/mol 23. 3'UTRs of human transcripts were used for piRNA target prediction.

### The TCGA data mining

The gene expression matrix and clinical information data of tumor and normal tissues were obtained from the Prostate Adenocarcinoma (PRAD) dataset in the TCGA database (https://portal.gdc.cancer.gov/). The TCGA-PRAD cohort comprising 52 normal patients and 501 tumor patients was used for analysis. Patients with no available clinical data were excluded from analysis in respective comparisons of the mRNA expression level in different subgroups. For the survival analysis, PCa patients with high and low mRNA expression were calculated by Kaplan-Meier analysis and a log-rank test.

### Animal xenograft model

LNCaP cells suspended in 100 µl 1:1 growth medium-Matrigel mixture were subcutaneously injected into the flank of intact male SCID mice aged six weeks. To study the effects of piR-4447944 on androgen-independent growth of implanted tumors, host mice bearing xenograft tumors were castrated by orchiectomy surgery when the tumor reached around 60 mm^3^. Castrated mice were randomly divided into the piR-4447944 agomir group and the negative control agomir (NC-agomir) group (5 per group). Afterwards, mice were administrated by intratumoral injection with 2 nmol of piR-4447944 agomir or NC-agomir in PBS every three days, respectively. Treatment was performed for twenty-six days, and the tumor volume was recorded every five days. The mice were sacrificed at the end of the experiment, and the xenografts were harvested and photographed. The tumor volume was calculated by length * width^2^/2 with a slide calliper. The cholesterol-modified piR-4447944 agomir and NC-agomir were commercially synthesized by Azenta Life Sciences (Shanghai, China). All animal experiments were performed in accordance with the university laboratory animal guidelines and with approval from the institutional animal experimentation ethics committee. The chemically modified piR-4447944 agomir sequence is as follows: 5'-UAAGCUAUCGGGCCCAUACCCAGAAAAUGUUGG-Cholesteryl-3' with 2'-OMe and phosphorothioate modification. The NC-agomir sequence is 5'-UUUGUACUACACAAAAGUACUG-Cholesteryl-3' with 2'-OMe and phosphorothioate modification.

### Statistical analysis

All experiments were performed in triplicates. The experimental data were analyzed using SPSS 22.0 (IBM, USA), GraphPad Prism 8.0 and the R package. Results were expressed as mean ± standard deviation (SD). Two-tailed student's t-tests were utilized to calculate statistical significance between two groups, and one-way ANOVA or two-way ANOVA was applied to compare three/more groups. Data in abnormal distribution were analyzed by non-parametric test. The survival curve was determined using Kaplan-Meier method and analyzed with the log-rank test. p < 0.05 was regarded as statistically significant. ns: non-significant, * p < 0.05, ** p < 0.01, *** p < 0.001, **** p < 0.0001.

## Results

### piR-4447944 is identified as a CRPC-related piRNA

**Figure 1:**
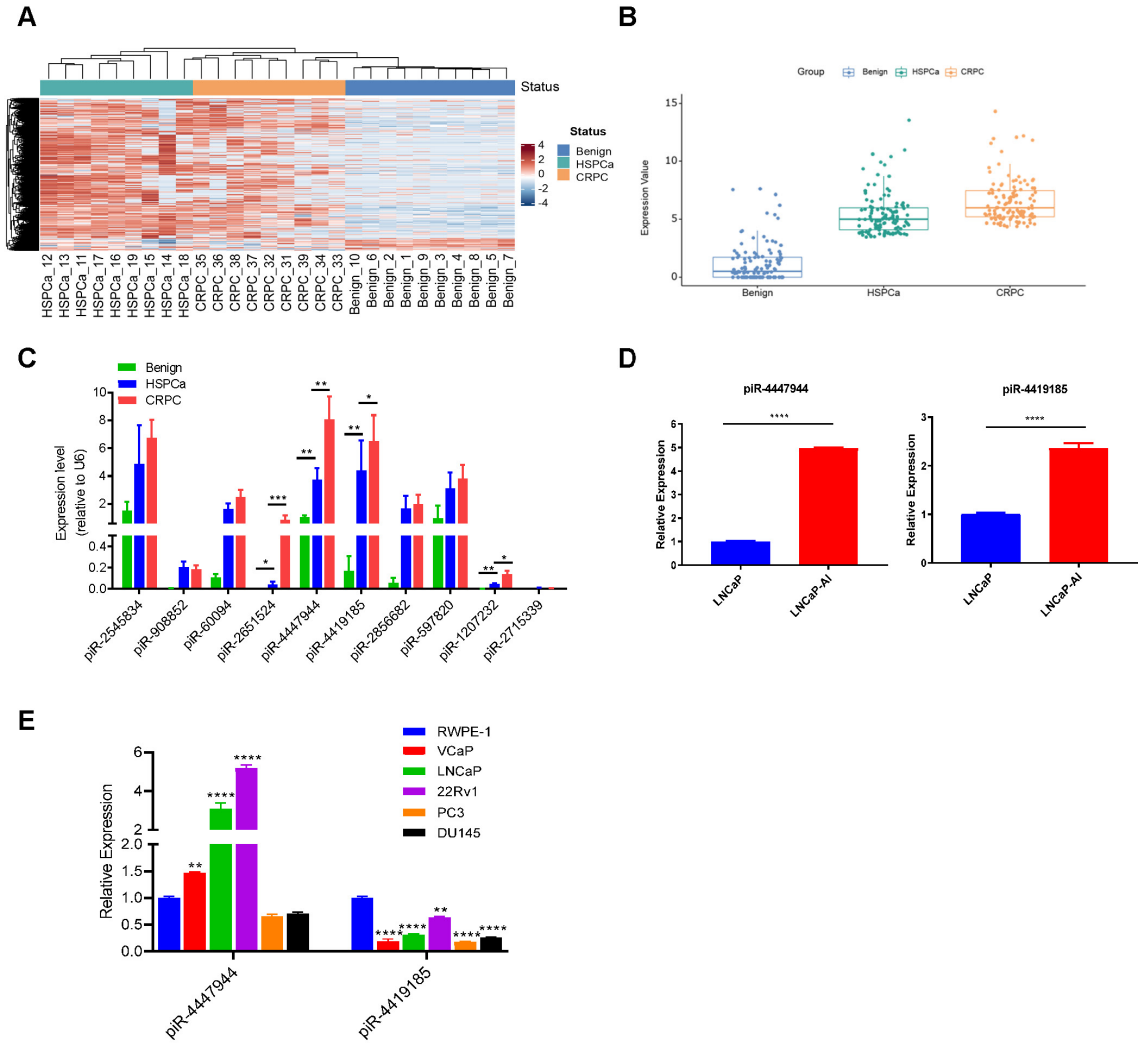
piR-4447944 is upregulated in CRPC tissues and cell lines. A, Small RNA sequencing revealed differentially expressed piRNAs among benign prostate tissues, HSPCa and paired CRPC tissues. The heatmap illustrated a distinguishable piRNA expression profiling among different groups. Red color indicates high expression and blue color indicates low expression. B, Box plot showed the expression levels of 117 significantly upregulated piRNAs in CRPC group, as compared with benign prostate group and HSPCa group. Each dot in one group indicated an individual piRNA and their expression levels were normalized to log2CPM (log2 counts per million). C, Expression levels of the top 10 highly expressed piRNAs in CRPC group were validated in a bigger cohort by RT-qPCR. Four piRNAs were validated to be significantly upregulated in the CRPC group compared to the HSPCa and benign prostate groups. D, The expression levels of two validated piRNAs in androgen independent LNCaP-AI cells were significantly higher than their parental hormone sensitive LNCaP cells. E, The expression levels of piR-4447944 and piR-4419185 were examined by qRT-PCR in human normal prostate epithelial cell line RWPE-1 and five human PCa cell lines (LNCaP, VCaP, 22Rv1, DU145 and PC3); data were normalized to RWPE-1 cells. Results are presented as mean ± SD. *p < 0.05; **p < 0.01, ***p < 0.001, ****p < 0.0001. Data were obtained from at least three independent experiments.

To identify CRPC-associated piRNAs, we first performed small RNA sequencing by analyzing 10 benign prostate tissues, 9 advanced/metastatic HSPCa tissues and their paired 9 CRPC tissues (Figure 1A and Figure S1). Our results discovered that 117 piRNAs were significantly up-regulated in CRPC tissues compared with benign and HSPCa tissues, displaying a step-wise increased expression pattern with a fold change ≥ 2.0 and an adjusted p-value<0.05 (Figure 1B and Figure S2). We further screened out the top 10 upregulated piRNAs according to their fold changes from the upregulated piRNAs in CRPC group (Table S7). RT-qPCR analysis was conducted to validate the expression changes of the above 10 piRNAs in a subset of 10 benign prostate tissues, 20 HSPCa tissues and 20 CRPC tissues. Then, four piRNAs (piR-2651524, piR-4447944, piR-4419185, piR-1207232) were validated to be significantly differentially expressed among CRPC, HSPCa and benign prostate tissues, with a higher expression level in CRPC (Figure 1C). In addition, we showed that the expression levels of piR-4447944 and piR-4419185 were significantly higher in androgen-independent CRPC cells LNCaP-AI than in its parental androgen-sensitive LNCaP cells (Figure 1D). Further comparison of piR-4447944 and piR-4419185 levels in various prostate-derived cell lines showed that piR-4419185 was expressed at significantly lower levels in all PCa cells than in non-malignant RWPE-1 cells, but the expression level of piR-4447944 in AR+ PCa cells was significantly higher than that in RWPE-1, and was comparable to RWPE-1 cells in AR- PCa cells (Figure 1E). Collectively, these data suggest that piR-4447944 might be a pivotal regulator in CRPC development.

To determine the growth response of the androgen-dependent PCa cell lines LNCaP and VCaP, and the androgen-independent PCa cell lines LNCaP-AI and 22Rv1, these cells were cultured in normal FBS medium or CS-FBS medium. As expected, VCaP cells and LNCaP cells were unable to grow in CS-FBS-medium, demonstrating their androgen-dependent characters. 22Rv1 cells also showed strong sensitivity to androgen deprivation, implying its androgen-responsive characteristics 24. Conversely, androgen-independent LNCaP-AI cells were able to grow in culture conditions with CS-FBS, confirming its CRPC characteristics (Figure S3).

### piR-4447944 promotes androgen-independent growth of PCa cells

Since piR-4447944 exhibited an increased expression in CRPC tissues and cells, we hypothesize that it might play a supportive role in CRPC growth. To elucidate this, we selected two androgen-sensitive PCa cell lines, LNCaP cells and 22Rv1 cells, both of which respond to androgen deprivation (Figure S3).

**Figure 2:**
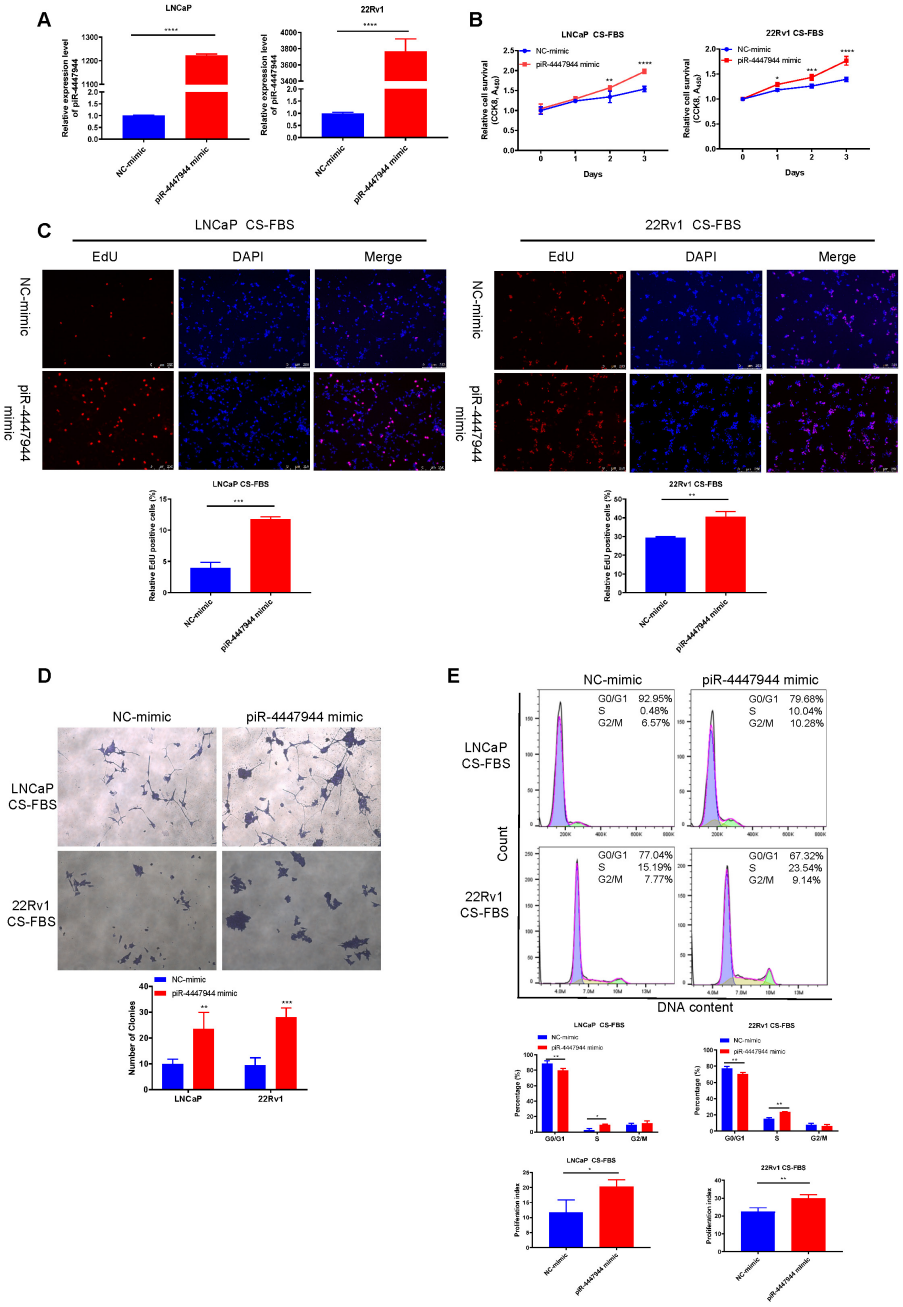
piR-4447944 promotes androgen-independent growth of PCa cells. A, Efficiency of piR-4447944 overexpression in PCa cells LNCaP and 22Rv1 by piRNA-mimic was verified by RT-qPCR. B, Effect of piR-4447944 overexpression on the cell viability of LNCaP and 22Rv1 cells under androgen-deprivation culture condition (CS-FBS) was determined by CCK8 assay. CS-FBS indicates charcoal-stripped fetal bovine serum, mimicking the androgen-deprivation conditions. C-D, The effect of piR-4447944 overexpression on EdU incorporation ability (C), colony formation ability (D) of LNCaP cells and 22Rv1 cells in androgen-deprived cultural medium. E, Cell cycle distribution of LNCaP and 22Rv1 cells transfected with NC-mimic or piR-4447944 mimic cultured in androgen-deprived cultural medium were detected by flow cytometry, and quantitative analysis of cell cycle distribution was shown in the bottom panel. The cell proliferation index was calculated as (S+G2/M)/(G0/G1+S+G2/M) x 100%. Results are presented as mean ± SD. *p < 0.05; **p < 0.01, ***p < 0.001, ****p < 0.0001. Data were obtained from at least three independent experiments.

We then first synthesized piRNA mimic that could overexpress piR-4447944 *in vitro* and then verified its overexpression efficiency in LNCaP cells and 22Rv1 cells by RT-qPCR (Figure 2A). *In vitro* growth analyses showed that when cultured in normal FBS conditions, piR-4447944-overexpressed PCa cells grew at a similar rate as the control group until Day-3 (Figure S4). However, overexpression of piR-4447944 exhibited enhanced growth ability and higher resistance to castration conditions (in CS-FBS medium), as determined by CCK8 and EdU incorporation assays (Figure 2B-C).

Consistently, piR-4447944-overexpressed PCa cells formed more and bigger colonies, as compared with control cells under the androgen-deprived conditions (Figure 2D). In addition, we found that piR-4447944 overexpression significantly increased cell population in the S phase and cell proliferation index, and reduced cell population in G0/G1 phase in LNCaP cells and 22Rv1 cells under CS-FBS culture conditions (Figure 2E). These data suggest that piR-4447944 plays a crucial role in the development of castration resistance in PCa.

### piR-4447944 enhances the migration and invasion, and reduces the apoptosis of PCa cells

**Figure 3:**
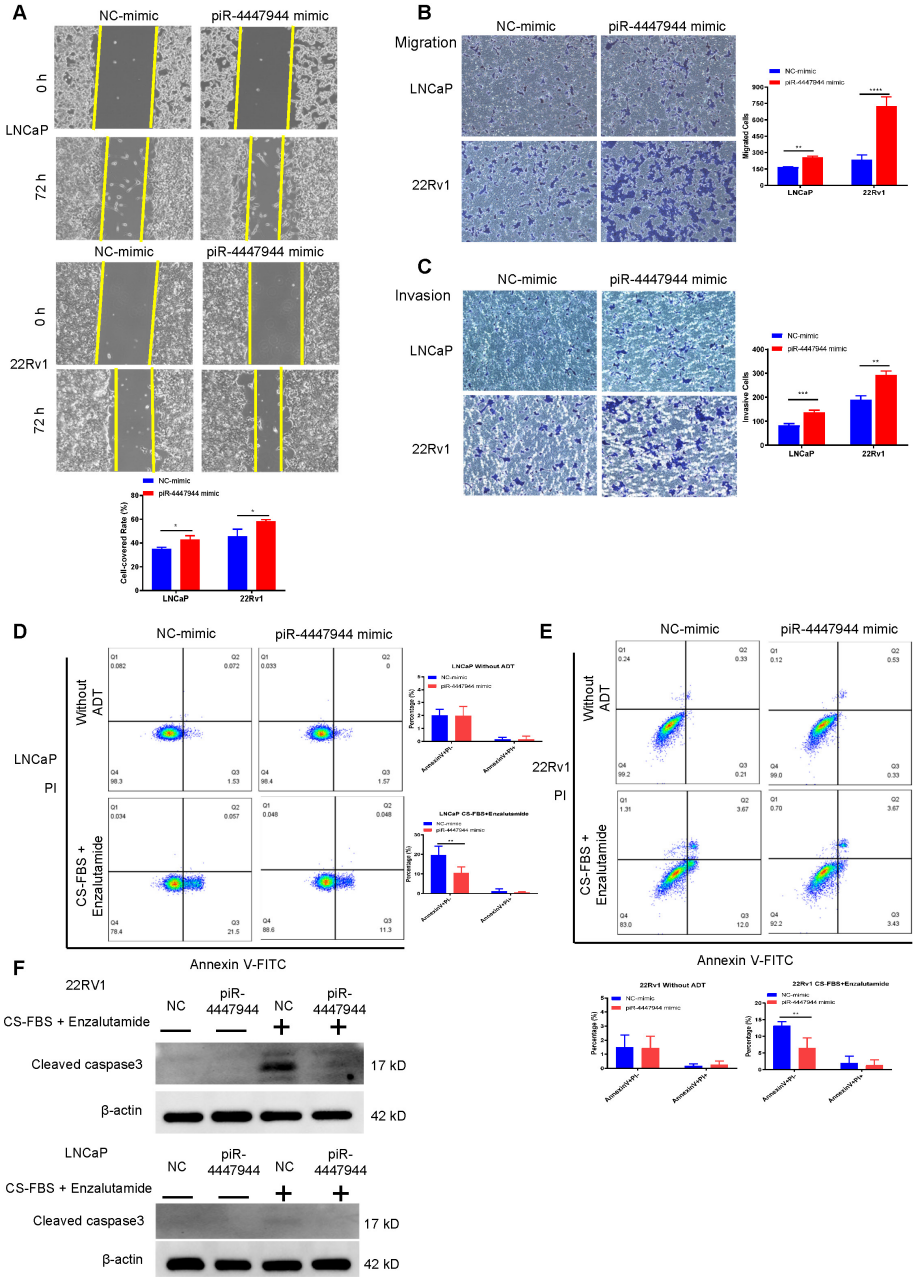
piR-4447944 promotes migration and invasion, and inhibits apoptosis of PCa cells. A, Wound-healing assay evaluated the migration ability of LNCaP cells and 22Rv1 cells with piR-4447944 overexpression. B-C, Transwell assays confirmed that the migration (B) and invasion (C) ability of PCa cells was enhanced with piR-4447944 overexpression. D-E, LNCaP cells (D) and 22Rv1 cells (E) transfected with NC or piR-4447944 mimic were cultured in an androgen-deprived medium for 48 h. The medium contained 10% CS-FBS and corresponding IC_50_ of enzalutamide (around 20 μM enzalutamide for LNCaP, and 50 μM enzalutamide for 22Rv1). Then apoptosis of PCa cells was evaluated by staining with Annexin V/PI, followed by flow cytometry analysis. The histogram showed the percentage (%) of early and late apoptotic cells from three independent experiments. F, Western blot detection of cleaved caspase 3 protein in control and piR-4447944-overexpressed LNCaP cells and 22Rv1 cells with or without ADT (medium containing 10% CS-FBS + the corresponding IC_50_ of enzalutamide). Results are presented as mean ± SD. *p < 0.05; **p < 0.01, ***p < 0.001, ****p < 0.0001. Data were obtained from at least three independent experiments.

We further assessed the impact of piR-4447944 on the migration and invasion abilities of PCa cells. As illustrated by the wound-healing assay in Figure 3A, piR-4447944 overexpression promoted the centripetal migration of PCa cells. The results of Transwell assays coincided with the wound-healing assay that forced piR-4447944 expression promoted the migration and invasion of LNCaP cells and 22Rv1 cells compared with the control groups (Figure 3B-C). We next subjected PCa cells cultured under androgen-deprived conditions to an apoptosis analysis. Enzalutamide, an androgen receptor antagonist frequently used in ADT, and the inhibitory effect of enzalutamide on LNCaP cells and 22Rv1 cells at different concentrations was detected by CCK8 assay (Figure S5). Treatment by a combination of 10% CS-FBS and the corresponding IC_50_ concentration of enzalutamide in the culture medium resulted in marked cell death of LNCaP and 22Rv1 cells, as evaluated by Annexin V-PI staining assay and cleaved caspase 3 immunoblotting detection (Figure S6, Figure 3D-F). In contrast, piR-4447944 overexpression significantly reduced the number of apoptotic PCa cells in response to ADT (Figure 3D-F). Collectively, our data revealed that the newly discovered piR-4447944 exerts a stimulating role in PCa migration and invasion, as well as apoptosis suppression.

### Repression of piR-4447944 inhibits LNCaP-AI cells proliferation, migration and invasion

**Figure 4:**
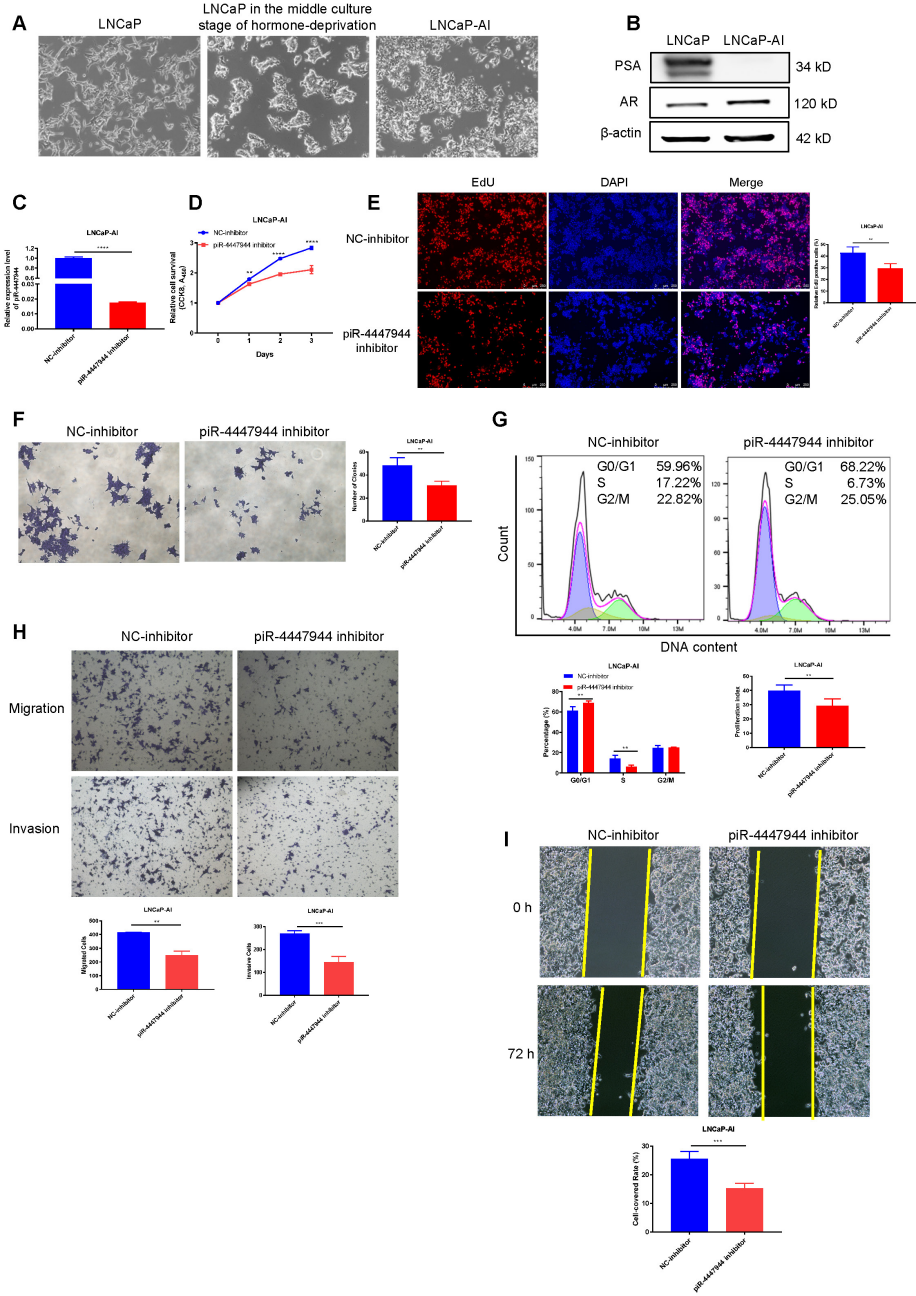
piR-4447944 suppression restores castration sensitivity and inhibits migration and invasion of LNCaP-AI cells. A-B, Identification of LNCaP-AI cells by cell morphology changes (A, magnification, 40x) and western blot (B). C, Knockdown efficiency of piR-4447944 inhibitor in LNCaP-AI cells was verified by RT-qPCR. D-F, Effect of piR-4447944 knockdown on LNCaP-AI cells viability in the androgen-deprived cultural medium was determined by CCK-8 assay (D), EdU incorporation assay (E) and colony formation (F). G, Representative flow cytometric histograms of PI staining of LNCaP-AI cells with control or piR-4447944 knockdown. Percentages of LNCaP-AI cells in the G0/G1, S and G2/M phases were quantified and shown in the bottom. The cell proliferation index was calculated as (S+G2/M)/(G0/G1+S+G2/M) x 100%. H-I, Influence of piR-4447944 knockdown on LNCaP-AI cells migration and invasion ability was evaluated by Transwell assays (H) and wound-healing assay (I). Results are presented as mean ± SD. *p < 0.05; **p < 0.01, ***p < 0.001, ****p < 0.0001. Data were obtained from at least three independent experiments.

The conversion of androgen-sensitive LNCaP cells into castration-resistant LNCaP-AI cells is similar to the pathogenesis of CRPC, so we established an androgen-independent CRPC cell line, LNCaP-AI, derived from the LNCaP cell line cultured in RPMI-1640 medium containing 10% CS-FBS. The initial morphology of LNCaP cells was a large cell body and long synapses, and the phenotype of LNCaP-AI cells exhibited shrinkage and rounding of the cell body and short synapses (Figure 4A). In contrast to LNCaP cells, LNCaP-AI cells proliferated persistently and maintained the ability to secrete PSA under androgen-depleted conditions (Figure S7). The AR protein level was significantly upregulated and PSA level was downregulated in LNCaP-AI cells when compared with parental LNCaP cells (Figure 4B), which is consistent with previous studies 25, 26. To further validate the significance of piR-4447944 in the development of CRPC, we evaluated the effect of piR-4447944 knockdown on androgen-independent LNCaP-AI cells. The knockdown efficiency of piR-4447944 inhibitor was verified by RT-qPCR (Figure 4C), and then the growth response of LNCaP-AI cells toward CS-FBS treatment was examined upon knockdown of piR-4447944. *In vitro* results showed that piR-4447944 suppression could inhibit the growth of LNCaP-AI cells and potentiate their sensitivity to androgen deprivation (Figure 4D-E). Consistent with cell growth results, inhibition of piR-4447944 in LNCaP-AI cells formed significantly fewer and smaller colonies, as compared with control cells (Figure 4F). Meanwhile, flow cytometry analysis showed that LNCaP-AI cells with piR-4447944 silencing increased cell population in G0/G1 phase, while reducing cell population in the S phase and cell proliferation index (Figure 4G). These data indicated that piR-4447944 knockdown could potentiate the sensitivity of CRPC cells to ADT.

Results of wound-healing and Transwell assays showed that, compared to the controls, inhibition of piR-4447944 significantly impeded the migration and invasion ability of LNCaP-AI cells as indicated by a lower rate of cells passing through the chamber and attenuated centripetal migration rate of LNCaP-AI cells (Figure 4H-I). Collectively, these data suggested an inhibitory role of piR-4447944 knockdown in CRPC cell proliferation, migration and invasion.

### piR-4447944/PIWIL2 complex post-transcriptionally regulates tumor suppressor NEFH

**Figure 5:**
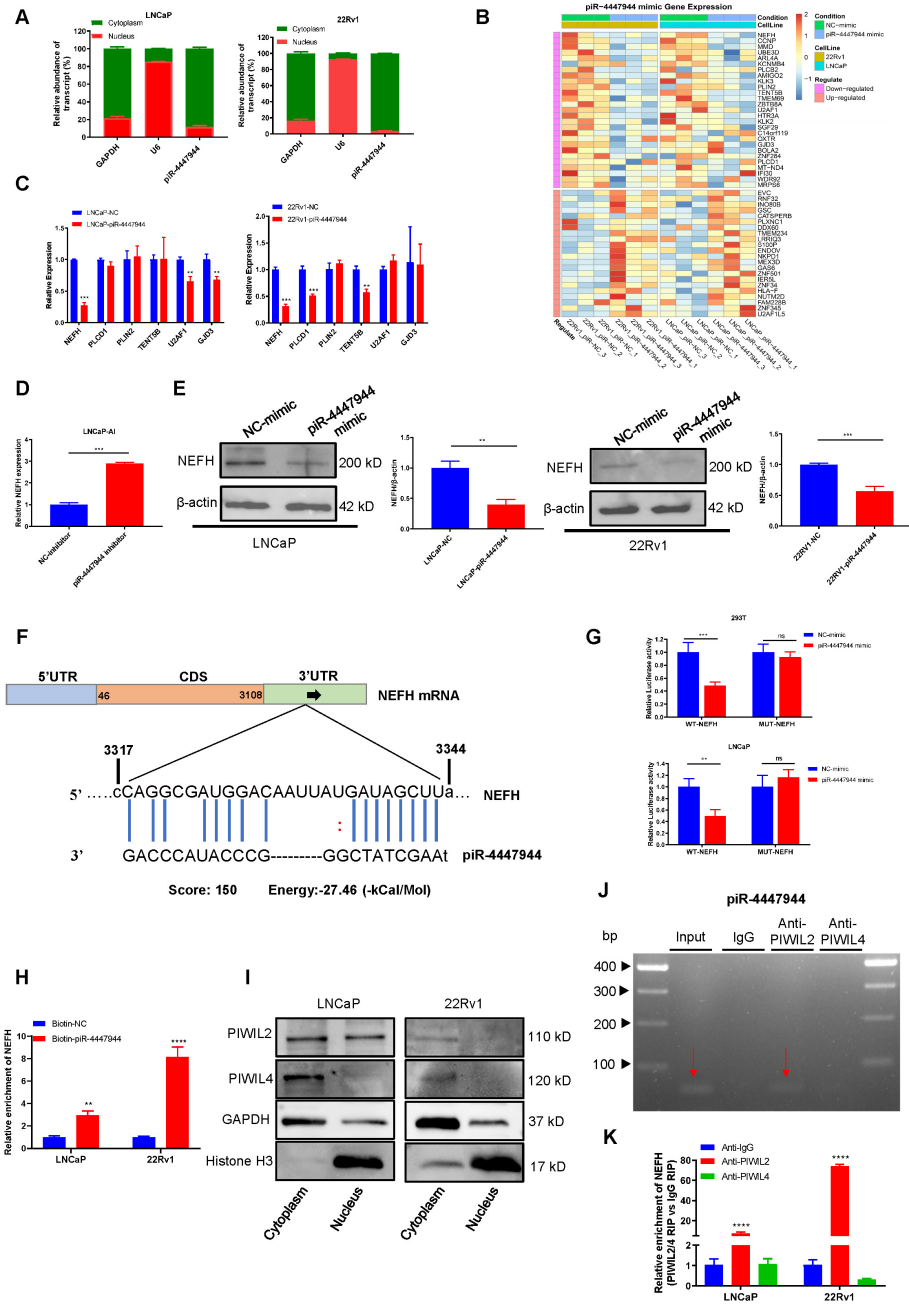
piRNA binds to PIWIL2 and inhibits tumor suppressor NEFH expression at the posttranscriptional level. A, Subcellular fractionation assay and real-time PCR detected the abundance of piR-4447944 in the nucleus and cytoplasm. GAPDH is the positive control for cytoplasm, and U6 is the positive control for the nucleus. B, Heatmap representing the expression levels of DEGs obtained from mRNA sequencing of LNCaP cells and 22Rv1 cells transfected with NC or piR-4447944 mimic. Each column represents the indicated sample, and each row indicates one DEG. Red and blue colors indicate high or low expression, respectively. The expression value is shown as a Z score of the normalized transcripts per million (TPM). C-D, Validation of the downregulated genes selected from mRNA sequencing results by RT-qPCR. The NEFH mRNA level was significantly decreased in piR-4447944-overexpressed PCa cells, while upregulated in LNCaP-AI cells with piR-4447944 knockdown. E, Western blot showed that piR-4447944 overexpression reduced NEFH protein levels in LNCaP and 22Rv1 PCa cells (left panel), and the quantification of three independent immunoblots was shown in the right panel. F, Potential target binding sites of piR-4447944 within the 3'UTR of NEFH were predicted by miRanda algorithm. G, Dual luciferase assay revealed a direct interaction between piR-4447944 and NEFH. H, RNA pull-down assay showed that NEFH was significantly enriched in the samples pulled down by the Biotin-piR-4447944 probe in LNCaP cells and 22Rv1 cells. I, Expression of PIWIL2 and PIWIL4 in the nuclear/cytoplasmic fraction of PCa cells was analyzed by western blot. J, Specific interaction of piR-4447944 and PIWIL2 protein in LNCaP cells was confirmed by RNA immunoprecipitation (RIP). The red arrow indicated the presence of piR-4447944 in the precipitate detected by electrophoresis after RT-PCR. K, RIP assays confirmed the interaction of PIWIL2 protein with NEFH transcript in LNCaP cells and 22Rv1 cells. Results are presented as mean ± SD. *p < 0.05; **p < 0.01, ***p < 0.001, ****p < 0.0001. Data were obtained from at least three independent experiments.

The subcellular localization of piRNAs is closely related to its biological function. As results of the subcellular fractionation assay (Figure 5A) and FISH assay (Figure S8) showed that piR-4447944 was predominately located in the cytoplasm fraction of LNCaP cells and 22Rv1 cells. And piRNAs have been well demonstrated to play crucial roles in cancer development by directing posttranscriptional regulation of target genes 15, 27-29. To further investigate the oncogenic mechanism of piR-4447944 in the development of CRPC, LNCaP cells and 22Rv1 cells transfected with NC or piR-4447944 mimic were cultured under androgen-deprived conditions, and then total RNA was extracted and subjected to mRNA sequencing to identify piR-4447944 target genes.

The fold changes of gene expression were calculated, and genes with log2|FC| > 0.2 and a corresponding p < 0.05 were considered differentially expressed. Notably, twenty-two upregulated and twenty-seven downregulated genes were found in the piR-4447944 overexpression group compared with the control group in both LNCaP cells and 22Rv1 cells (Figure 5B, Figure S9). From these downregulated targets, we chose six genes based on their previously reported roles in cancers and further validated them in PCa cells by RT-qPCR (Figure 5C). Among these candidate targets, NEFH stands out because only NEFH was downregulated by piR-4447944 in both LNCaP cells and 22Rv1 cells, and further knockdown of piR-4447944 in LNCaP-AI cells increased NEFH expression levels (Figure 5C-D). Western blot indicated that NEFH protein level was significantly reduced in piR-4447944-overexpressed PCa cells (Figure 5E).

Then the potential binding sites of piR-4447944 within the 3'UTR of NEFH were analyzed by miRanda algorithm, as shown in Figure 5F. Next, a dual-luciferase reporter assay was performed using luciferase reporter plasmids with WT or MUT of NEFH to explore the potential interaction between piR-4447944 and NEFH. After transfection with piR-4447944 mimic, the luciferase activities of WT-NEFH group were significantly reduced compared with cells transfected with NC-mimic, while no significant differences were detected in the MUT-NEFH group (Figure 5G, Figure S10). To further verify the interaction between piR-4447944 and NEFH, RNA pulldown assay was performed in LNCaP cells and 22Rv1 cells, followed by RT-qPCR. Results demonstrated significantly specific NEFH enrichment in PCa cells transfected with biotin-labeled piR-4447944 compared with that in control (Figure 5H). PiRNAs located in the cytoplasm have been reported to be involved in post-transcriptional gene suppression by forming piRNA/PIWI complexes 10. It was found that PIWIL2 and PIWIL4, two major members of the PIWI gene family in PCa, were predominantly expressed in the cytoplasm of PCa cells (Figure 5I). The specific interaction of piR-4447944 and PIWI proteins was verified in LNCaP and 22Rv1 cells by RIP, and results showed that piR-4447944 could bind to PIWIL2 but not PIWIL4 (Figure 5J, Figure S11). Additionally, RIP results using anti-PIWIL2 or anti-PIWIL4 antibodies further showed that NEFH was pulled down more abundantly in the PIWIL2 group than in the IgG group, while no significant enrichment was detected in the PIWIL4 group (Figure 5K). Taken together, these data suggest that tumor suppressor NEFH is negatively regulated by the piR-4447944/PIWIL2 complex and serves as its direct target.

### Restoration of NEFH inhibits piR-4447944-mediated androgen-independent growth of PCa cells

**Figure 6:**
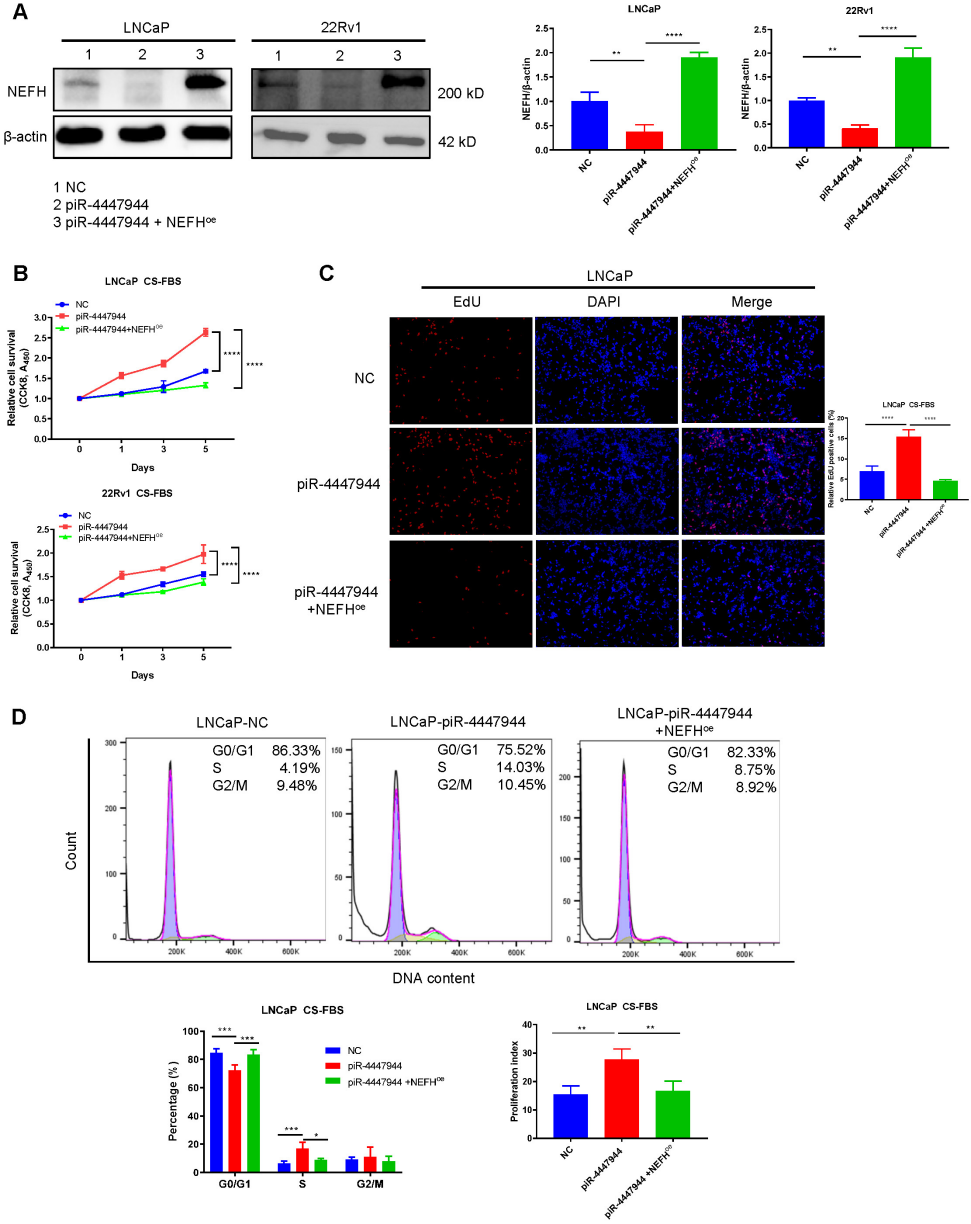
piR-4447944 promotes androgen-independent growth of PCa cells by targeting NEFH. A, The NEFH protein level was restored by co-transfection with NEFH-overexpression (NEFH^oe^) plasmid in piR-4447944-overexpressed LNCaP cells and 22Rv1 cells. Statistical histograms were shown in the right panel. B-D, Restoration of NEFH expression attenuated the enhanced androgen-independent growth of PCa cells induced by piR-4447944 under CS-FBS conditions, as indicated by CCK8 assay (B), EdU incorporation assay (C) and cell cycle assay (D). The cell proliferation index was calculated as (S+G2/M)/(G0/G1+S+G2/M) x 100%. Results are presented as mean ± SD. *p < 0.05; **p < 0.01, ***p < 0.001, ****p < 0.0001. Data were obtained from at least three independent experiments.

To further explore the clinical importance of NEFH in PCa, we first analyzed the relationship between NEFH expression levels and tumor clinical characteristics in the TCGA-PRAD dataset. The downregulation of NEFH was validated in PCa tissues, and reduced NEFH level was significantly more pronounced in PCa with advanced T stage and poor differentiation (higher Gleason score) (Figure S12). Kaplan-Meier survival analysis indicated that PCa patients with low NEFH expression had a trend towards worse prognosis in terms of overall survival (OS, p=0.054) than those with high NEFH expression (Figure S12). We next investigated whether the increased androgen-deprivation resistance activity of piR-4447944 depended on the alteration of NEFH. To this end, we first examined the efficiency of NEFH overexpression in piR-4447944-transfected LNCaP cells and 22Rv1 cells, and verified that co-transfection with NEFH-vector restored NEFH expression (Figure 6A). CCK8 assay revealed that piR-4447944 overexpression promoted PCa cells proliferation under CS-FBS conditions, while restoration of NEFH in piR-4447944-transfected PCa cells attenuated cell proliferation (Figure 6B). EdU incorporation assay corroborated the CCK8 data that overexpression of NEFH attenuated the increased resistance to androgen deprivation in piR-4447944-transfected PCa cells (Figure 6C, Figure S13). To further examine cell cycle changes of PCa cells in the androgen-deprived environment, we performed flow cytometry and found that NEFH overexpression significantly reduced the piR-4447944-induced increases in S phase and cell proliferation index of PCa cells (Figure 6D, Figure S14). The above results indicated that piR-4447944 promotes androgen independence of PCa cells by targeting NEFH.

### piR-4447944 overexpression promotes castration-resistant growth of PCa *in vivo*

**Figure 7:**
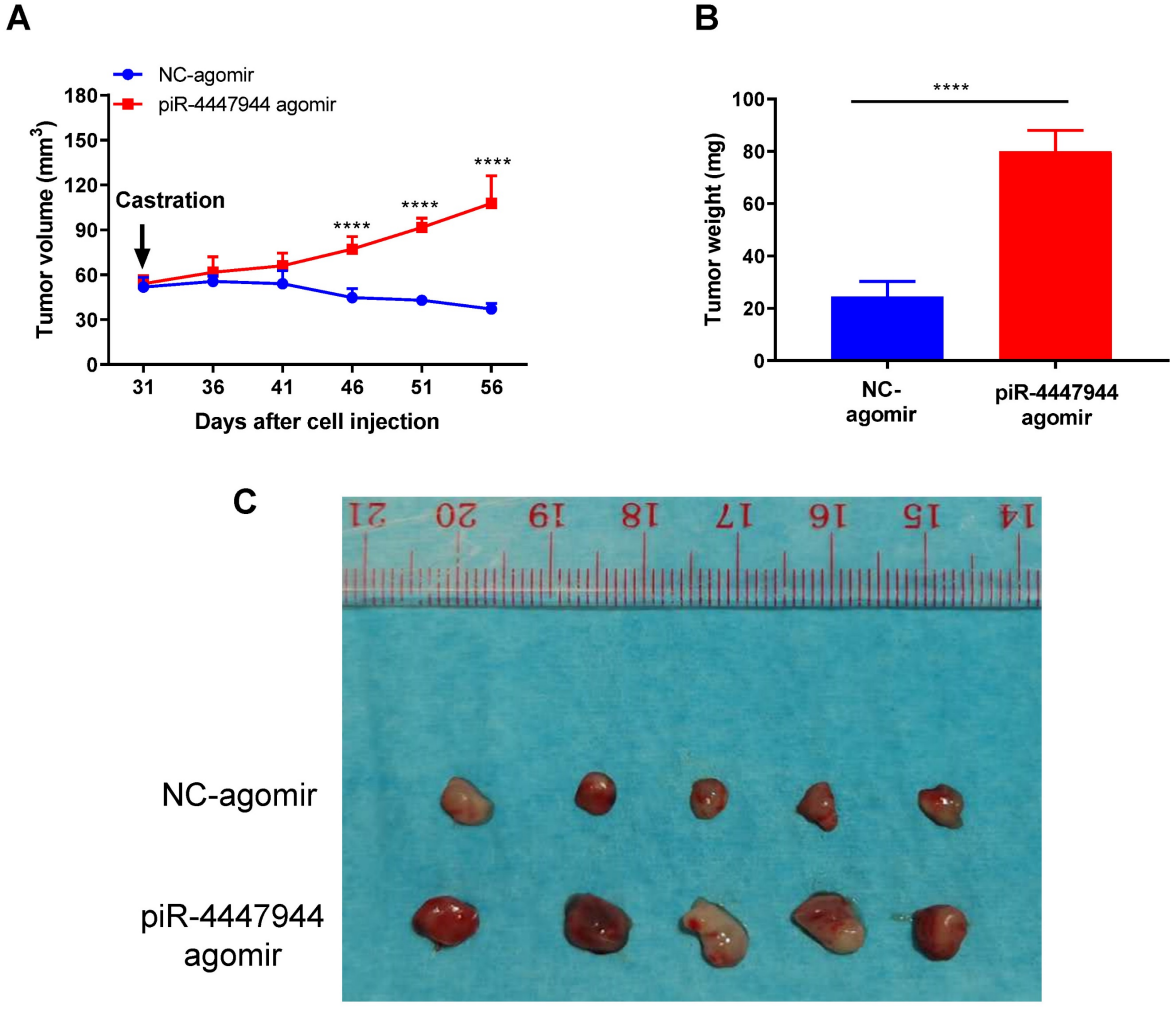
piR-4447944 accelerates *in vivo* castration-resistant growth capacity of LNCaP-derived xenograft tumors. A, Effect of piR-4447944 on the growth of subcutaneous tumor xenografts in the castrated SCID mice (n=5). Tumors treated with piR-4447944 agomir continued to grow aggressively, whereas tumors treated with NC-agomir ceased to grow. B, Weight of tumor xenografts in the castrated hosts was measured after the tumors were surgically dissected (n=5). piR-4447944 agomir group formed heavier tumors than the control group. C, Image showed the dissected xenografts tumors in each group. Results are presented as mean ± SD. *p < 0.05; **p < 0.01, ***p < 0.001, ****p < 0.0001.

To further validate the enhanced resistance to androgen deprivation exerted by piR-4447944 in PCa cells, we assessed the impact of forced piR-4447944 expression in castrated SCID mice bearing xenograft tumor derived from LNCaP cells. As a result, piR-4447944-overexpressed xenograft tumors showed no response to castration and continued to grow, in sharp contrast to the control group that stopped growing in the castrated hosts (Figure 7A). We observed a significant increase in the tumor weight and volume of xenografts treated with piR-4447944-agomir in the castrated host compared with those treated with NC-agomir (Figure 7B-C). The above results indicated that piR-4447944 accelerated androgen-independent growth of tumor xenografts in SCID mice and promoted the development of CRPC *in vivo*.

## Discussion

Overcoming castration resistance is a conundrum in the clinical treatment of PCa. Fortunately, with the development of high-throughput sequencing technology in recent years, the number of newly identified targets associated with CRPC progression is growing fast, shedding new insights for curing CRPC. Due to the activation of multiple pathways in CRPC, it may be insufficient to rely on a single therapeutic approach to address the lethal phenotype of CRPC effectively. Compared with protein-coding genes and other types of ncRNAs, piRNAs are emerging as novel therapeutic targets in cancer treatment 10, 30. Certain piRNAs have demonstrated the capacity to manipulate the expression of multiple components within a signaling pathway or cellular process 31-33. Numerous studies to date have also revealed a close correlation between piRNA expression and human cancers, including PCa 30, 34. In addition, certain piRNAs have shown great promise as diagnostic or prognostic markers in cancers 30, 35. However, the functions of piRNAs in CRPC development remain unclear. In this study, we performed small RNA sequencing to screen out an uncharacteristic and upregulated piRNA named piR-4447944 in CRPC. Additionally, we investigated the function and mechanism of piR-4447944 during CRPC development. We showed for the first time that piR-4447944 is upregulated in CRPC tissues and cells compared with androgen-sensitive PCa tissues and cells, and enforced piR-4447944 expression promotes androgen-independent growth of PCa significantly both *in vitro* and *in vivo*. Furthermore, our functional experiments provide convincing evidence to support the association of piR-4447944 with an aggressive clinical phenotype, where piR-4447944 promotes PCa cells migration, invasion and suppression of apoptosis.

Although piRNAs were initially identified in mammalian germlines, a growing number of studies have revealed that piRNAs are widely expressed and play essential roles in somatic cells 8, 36. Aberrantly expressed piRNAs have been discovered to exert tumor-promoting or tumor-suppressor effects in various cancers 17, 30. In addition, studies showed that certain piRNAs are involved in somatic gene regulation through epigenetic mechanisms, such as transcriptional gene silencing and DNA methylation at CpG sites in genome 37-39. On the other hand, piRNA-guided PIWI-protein complexes are involved in post-transcriptional gene regulation to degrade their targeted RNAs through a miRNA and/or siRNA-like mechanism. For example, piR-55490 inhibits lung tumor proliferation by binding to the 3'UTR of mTOR mRNA and degrading mTOR mRNA 15. piR-39980, a fibrosarcoma tumor suppressor, inhibits ribonucleoside-diphosphate reductase subunit M2 (RRM2) expression by binding to its 3'UTR, thus exerting an anti-tumor effect 40. Moreover, piR-30188 binds to a lncRNA OIP5-AS1 and inhibits its expression, leading to the suppression of glioma cell malignant phenotype via the miR-367/CEBPA/TRAF4 pathway 16. To further understand the mechanic role of piR-4447944 in CRPC, we interrogated its potential downstream targets. In this study, we first found that piR-4447944 and PIWIL2 are mainly located in the cytoplasm of PCa cells, and piR-4447944 can bind to PIWIL2 to form PIWIL2/piR-4447944 complex.

Notably, PIWIL2, a member of the PIWI protein subfamily, is widely expressed in tumors and leads to cancer progression 41-44. In PCa, PIWIL2 expression levels were positively correlated with the Gleason score (assessment of cancer aggressiveness by histology) and the TNM (tumor node metastasis) stage of tumors 44. Hence, we further explored the post-transcriptional regulatory mechanism of the PIWIL2/piR-4447944 complex in regulating the expression of specific downstream targets in PCa. By mRNA sequencing, NEFH is identified to be regulated by piR-4447944, and further RT-qPCR and western blot demonstrated that piR-4447944 could inhibit NEFH expression both at the mRNA and protein levels. We predicted that piR-4447944 could target the 3'UTR region of NEFH through base-pairing by miRanda algorithm (Figure 5F). And results of dual luciferase assay, RNA pull-down and RIP assays further provided convincing evidence for the direct interaction between PIWIL2/piR-4447944 complex and downstream target NEFH.

NEFH is a key subunit of the neurofilament complex that has been reported to be involved in a variety of cancers 45-49. NEFH is functionally identified to exhibit attributes of a tumor suppressor, as knockdown experiments revealed increased tumorigenicity and promotion of tumor progression, while overexpression of NEFH was associated with decreased cell growth 46, 47, 50. Calmon et al. reported that the inactivation of NEFH promoted an aggressive phenotype in breast cancer, whereas the re-expression of NEFH was shown to suppress its malignant biological behavior 46. A study by Kim et al. showed that esophageal cancer cells with restored NEFH expression displayed reduced proliferation ability 47. Schleicher et al. revealed the downregulated NEFH mRNA and protein expression levels in PCa as early as 1997 51. A recent study by Wang et al. showed that overexpression of NEFH significantly inhibited cell growth, colony formation and DNA replication ability of CRPC cell line, and promoted apoptosis, confirming that NEFH has a tumor suppressor effect on PCa 52. In addition, Wang et al. showed that the NEFH mRNA level was notably reduced in metastatic PCa tissues compared with localized PCa and benign tissues from a microarray dataset GSE21032. And decreased NEFH expression was associated with a higher risk of biochemical recurrence in PCa patients 52, 53. In this study, we showed that restoration of NEFH abrogated the tumor-promoting effects in an androgen-deprived environment induced by piR-4447944. Taken together, these findings suggest a posttranscriptional regulation mechanism by piR-4447944/PIWIL2 in the development of CRPC by repression of NEFH.

Loss of NEFH leads to increased invasiveness in multiple cancers 46, 47. In addition, NEFH hypermethylation was also found to be associated with adverse clinical parameters, such as advanced tumor stage and distant metastasis in cancer patients 46, 48. The effect of NEFH silencing on the malignant invasive behavior of tumors, possibly through the involvement of neurofilaments in the reorganization of the cytoskeleton, resulting in higher motility and ability to migrate and invade into neighboring tissues due to the disturbance of the rearrangements of cytoskeleton and the dynamic deformation of cells. In our study, we identified that piR-4447944 was an upstream regulator of NEFH. Considering the significant role of NEFH in cell motility, it may participate in the piR-4447944-mediated PCa cells dissemination.

In summary, our study demonstrates for the first time that piR-4447944 is essential for prostate tumor-propagating cells and mediates the androgen-independent growth of PCa. Further mechanism exploration identified that the tumor suppressor NEFH is directly inhibited by piR-4447944 in PCa cells. *In vitro* and *in vivo* results showed that anti-sense RNA against piR-4447944 may be a promising approach to reduce prostate tumor dissemination and delay CRPC progression. Our study sheds new insights into the regulation of CRPC by a small non-coding piRNA, which may form a basis for CRPC treatment.

## Supplementary Material

Supplementary figures and tables.

## Abbreviations

ADT: androgen deprivation therapy
AI: androgen independent
AR: androgen receptor
CRPC: castration-resistant prostate cancer
CS-FBS: charcoal stripped fetal bovine serum
CPM: counts per million
DEGs: differentially expressed genes
DHT: dihydrotestosterone
FBS: fetal bovine serum
FFPE: formalin-fixed paraffin-embedded
FISH: fluorescence *in situ* hybridization
HSPCa: hormone-sensitive prostate cancer
IC_50_: half maximal inhibitory concentration
MUT: mutant-type
NC: negative control
ncRNAs: non-coding RNAs
NEFH: neurofilament heavy polypeptide
OS: overall survival
PBS: phosphate buffered saline
PCa: prostate cancer
piRNAs: piwi-interacting RNAs
PSA: prostate-specific antigen
RIP: RNA immunoprecipitation
TNM: tumor node metastasis
TPM: transcripts per million
TURP: transurethral resection of the prostate
UMI: unique molecular identifiers
WT: wild-type
