# Supplementary material for: PiRNA-4447944 promotes castration-resistant growth and metastasis of prostate cancer by inhibiting NEFH expression through forming the piRNA-4447944-PIWIL2-NEFH complex
Source: Int J Biol Sci. 2024 Jul 1;20(9):3638–55. doi: 10.7150/ijbs.96173 (PMC11234203; doi:10.7150/ijbs.96173)
Supplement: Supplementary file 1 — Supplementary figures and tables. [file ijbsv20p3638s1.pdf]

Supplementary Figures

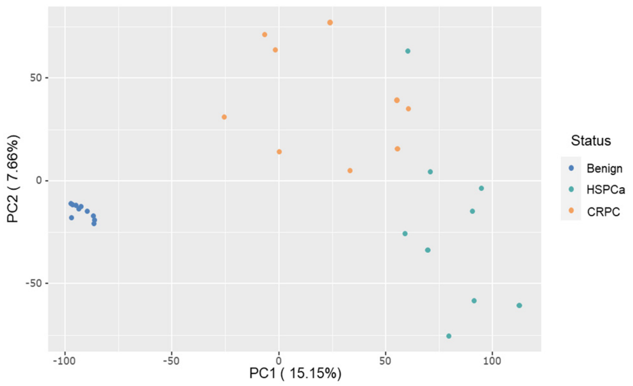

Figure S1: Principal component analysis (PCA) was performed on the small RNA sequencing data according to the expression levels of piRNAs in different groups of tissue samples, including benign, HSPCa and CPRC.

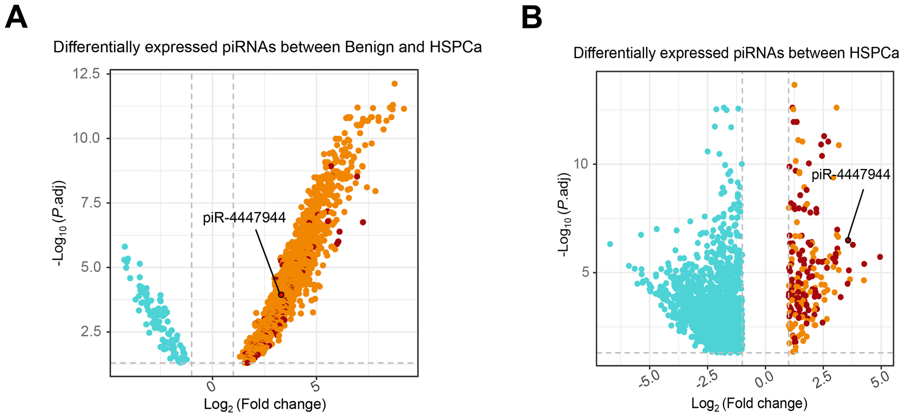

Figure S2: Volcano plot of differentially expressed piRNAs in HSPCa vs. Benign (A) and CRPC vs. HSPCa (B). The horizontal dashed line represents the adj. p-value cutoff (0.05) for differentially expressed piRNAs. The vertical dashed line represents the fold change cutoff (⩾2 or ⩽-2) of differentially expressed piRNAs. Upregulated piRNAs are indicated in orange, downregulated piRNAs are indicated in cyan. The simultaneously upregulated piRNAs in CRPC vs HSPCa (A) or HSPCa vs Benign (B) are represented in red.

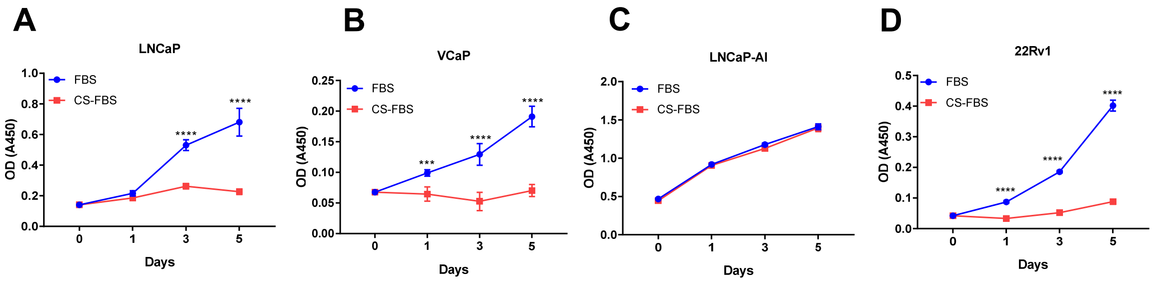

Figure S3: The characters of PCa cell lines under androgen-deprived conditions (in 10% CS-FBS medium) or normal culture conditions (in 10% FBS medium) were detected by cell viability assay. A-B, Proliferation rates of LNCaP cells and VCaP cells were significantly inhibited under androgen-deprived conditions, which demonstrated significant androgen-dependent character. C, LNCaP-AI cells were still able to grow in an androgen-deprived environment, validating its castration-resistant character. D, 22RV1 cells showed strong sensitivity to androgen-deprivation, implying its androgen-responsive character.

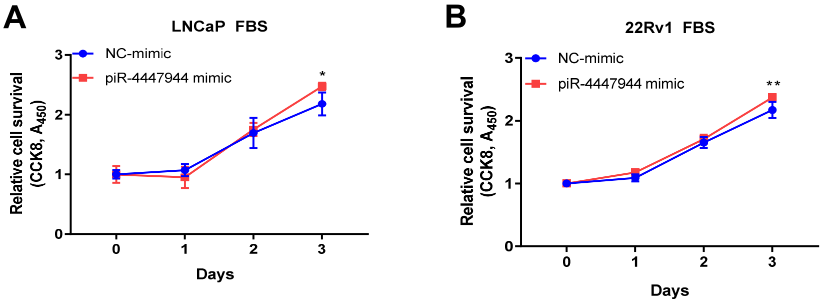

Figure S4: Effect of piR-4447944 overexpression on cell viability of LNCaP cells (A) and 22Rv1 cells (B) under normal culture condition (10% FBS) was determined by CCK8 assay.

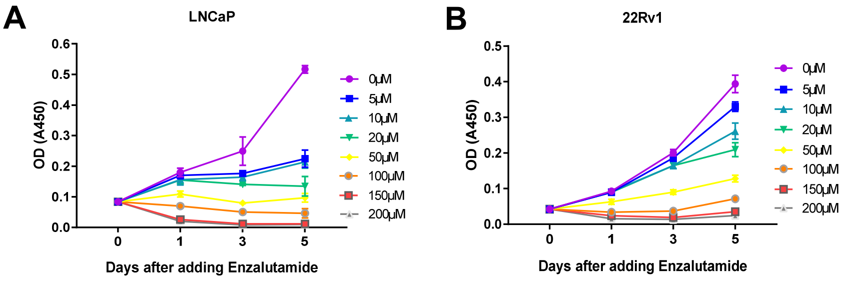

Figure S5: Effect of enzalutamide treatment on cell viability of LNCaP cells (A) and 22Rv1 cells (B). PCa cells were treated with a dose range of 5 μM to 200 μM enzalutamide or 0 μM (DMSO). Cell viability was evaluated at indicated post-treatment time by CCK8 assay.

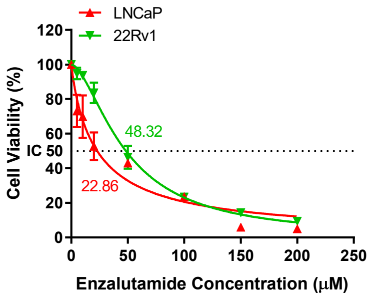

Figure S6: Calculation of the IC_{50} of enzalutamide in LNCaP cells and 22Rv1 cells for 48 h. The IC_{50} value was calculated using a nonlinear regression analysis in GraphPad Prism 8.0.

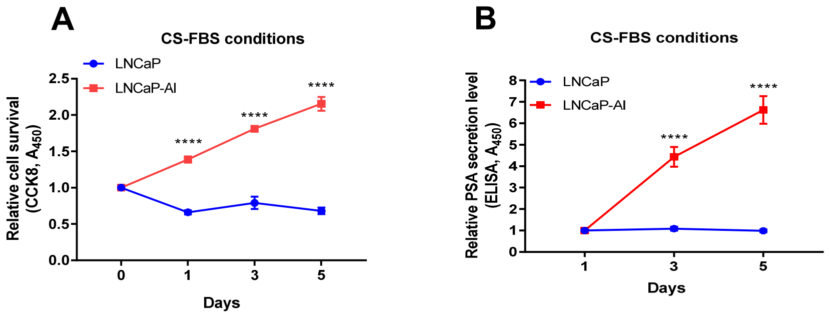

Figure S7: Biological characteristic differences between LNCaP and LNCaP-AI cells. A, In androgen-deprived medium, the proliferation of LNCaP-AI cells was significantly higher than that of LNCaP cells. B, In androgen-deprived medium, the PSA secretion ability of LNCaP-AI cells was not affected, but the PSA secretion of LNCaP cells was significantly inhibited.

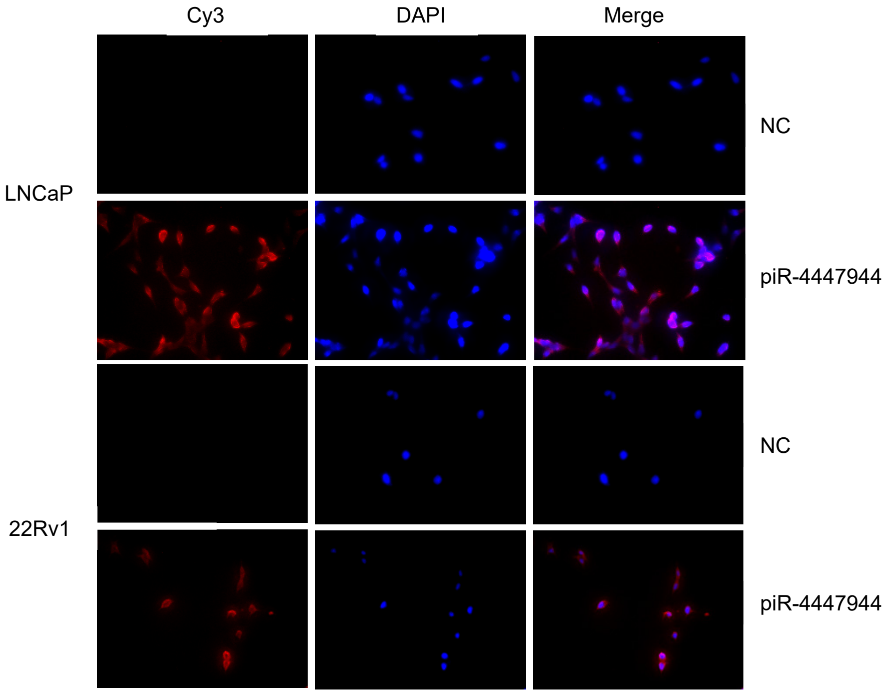

Figure S8: FISH assay shows that piR-4447944 is mainly distributed in the cytoplasm of LNCaP cells and 22Rv1 cells. DAPI stains the nucleus in blue, and the probe label of piR-4447944 is labeled with Cy3 and stains red. NC, negative control.

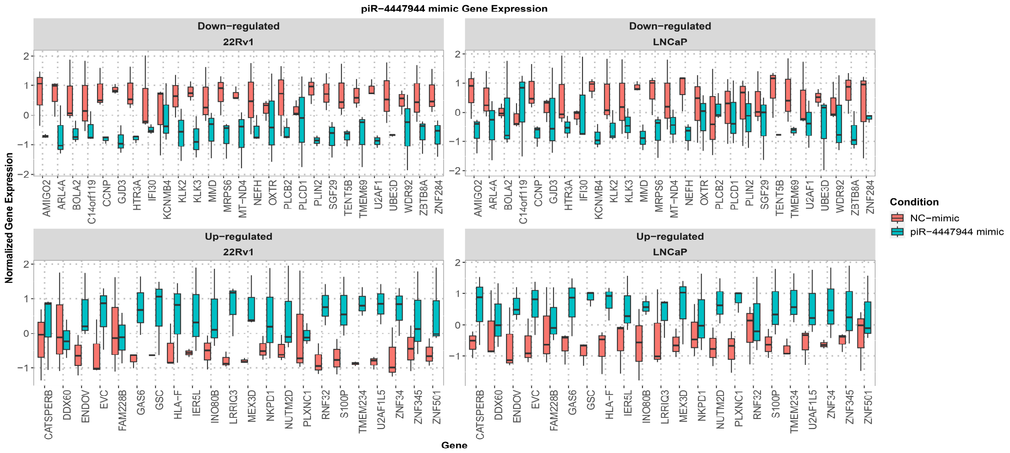

Figure S9: Boxplot depicting the expression values of DEGs between piR-4447944 overexpression group and control group in LNCaP cells and 22Rv1 cells based on the mRNA sequencing results. Expression value shown as a Z score of the normalized TPM.

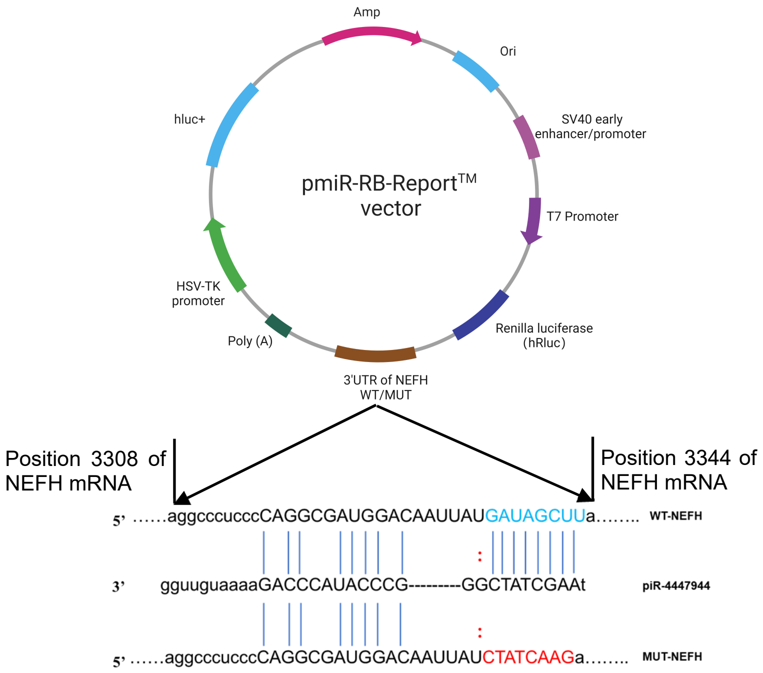

Figure S10: Schematic representation of wild-type (WT) and mutant (MT) NEFH 3′UTR luciferase reporter constructs. The piR-4447944 wild-type binding sites within the 3′UTR of NEFH were indicated in blue, and the mutant binding sites in red. This graph created by Biorender.com.

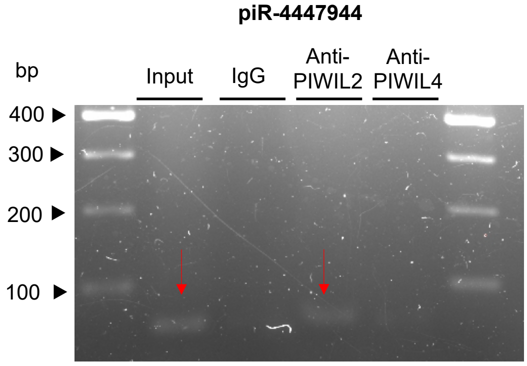

Figure S11: Specific interaction of piR-4447944 and PIWIL2 protein in 22Rv1 cells was confirmed by RIP using anti-PIWIL2 or anti-PIWIL4 antibodies, and rabbit IgG was used as a control antibody. Red arrow indicated the presence of piR-4447944 in the precipitate detected by electrophoresis on 3% agarose gel after RT-PCR.

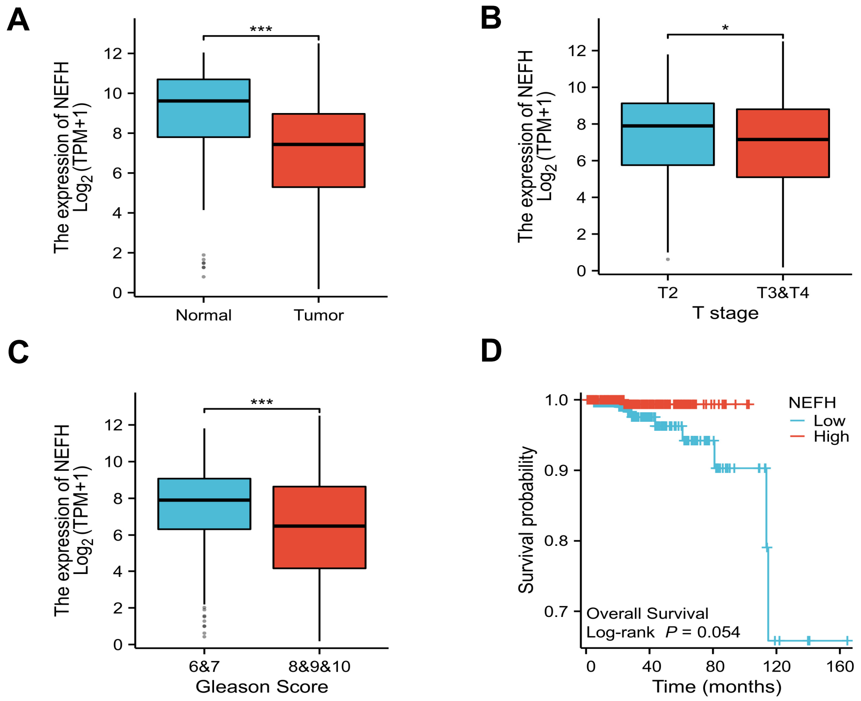

Figure S12: Low NEFH expression correlates with advanced disease and poor prognosis in PCa. A, NEFH was expressed at a lower level in PCa tissues than in normal prostate tissues. B-C, Low NEFH expression was associated with advanced T stages and poor differentiation (high Gleason score) in PCa. D, Low-NEFH expression group showed a strong tendency to be associated with poor OS in PCa (p=0.054).

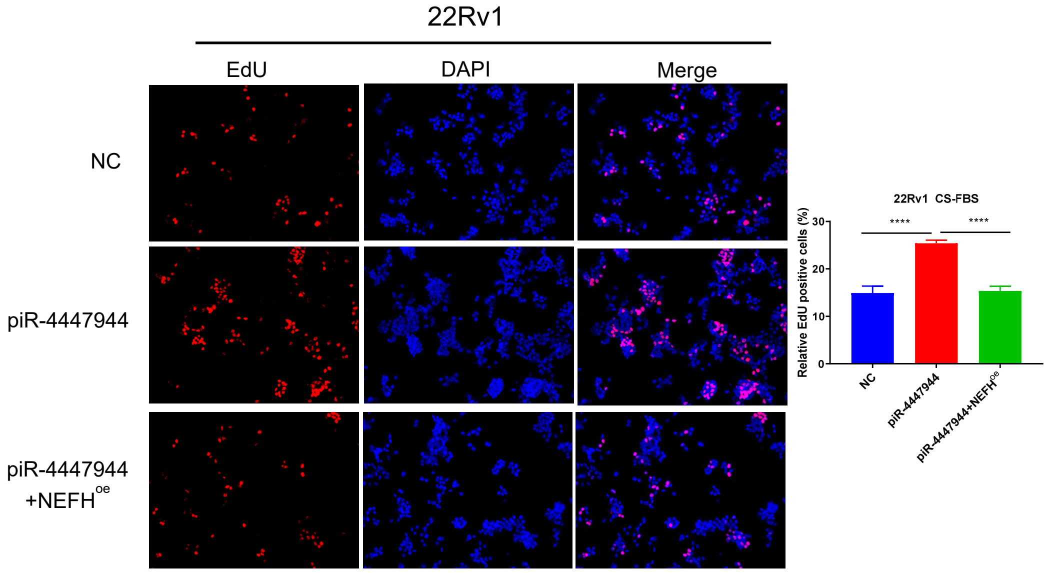

Figure S13: EdU incorporation assay validated that restoration of NEFH expression attenuated the proliferation ability of piR-4447944-transfected 22Rv1 cells in an androgen-deprivation environment.

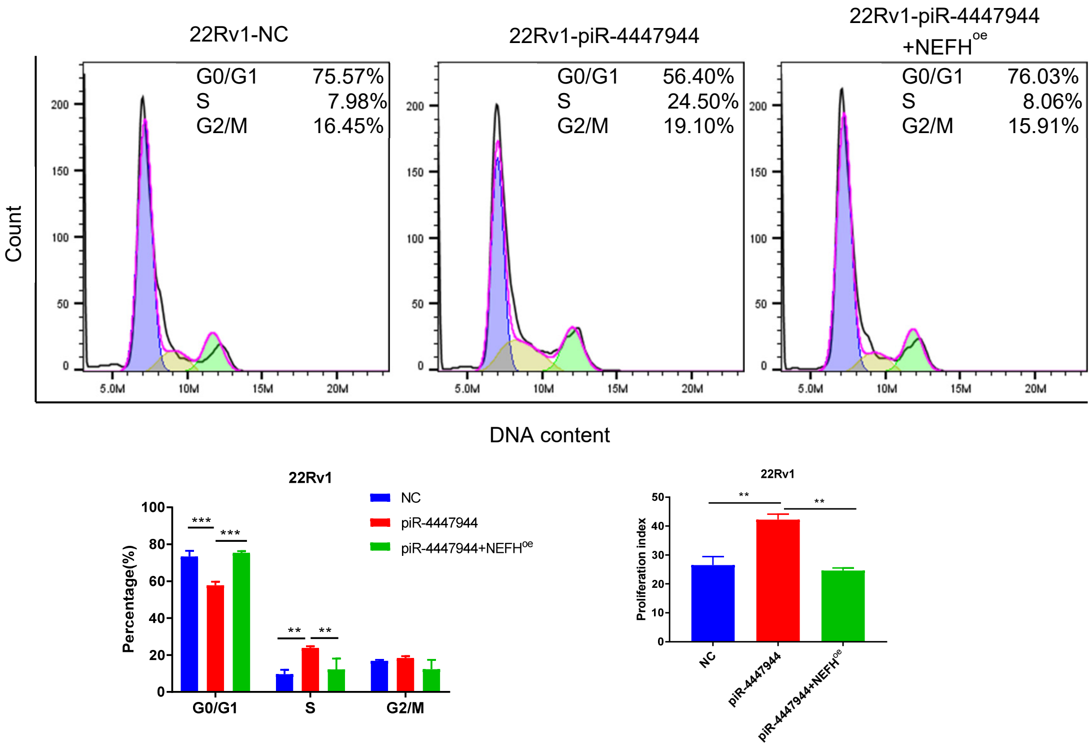

Figure S14: Cell cycle assay showed that NEFH overexpression induced cellular S-phase arrest and reduced cell proliferation index in piR-4447944-overexpressed 22Rv1 cells under androgen-deprived conditions. Cell proliferation index was calculated as (S+G2/M)/(G0/G1+S+G2/M) x 100%.

Supplementary Tables

Table S1: Clinical features of patient cases in this study

Patient Number	Age	Gleason Score	Stage	Tumor Type	PSA (ng/ml)	TNM Stage	Treatment	Sample type	Biopsy date/TURP date
1	63			Benign	3.92			Fresh sample from Biopsy	07/10/2020
2	73			Benign	8.4			Fresh sample from Biopsy	21/10/2020
3	62			Benign	7.4			Fresh sample from Biopsy	11/11/2020
4	60			Benign	8.2			Fresh sample from Biopsy	25/11/2020
5	66			Benign	7.7			Fresh sample from Biopsy	25/11/2020
6	58			Benign	5.3			Fresh sample from Biopsy	14/12/2020
7	71			Benign	5.4			Fresh sample from Biopsy	14/12/2020
8	71			Benign	7.7			Fresh sample from Biopsy	21/10/2020
9	70			Benign	9			Fresh sample from Biopsy	04/11/2020
10	66			Benign	5			Fresh sample from Biopsy	11/11/2020
11	79	4+4	IV	HSPCa	51.2	T4N0M0	GnRH agonists	FFPE sample from Biopsy	15/01/2020
12	75	4+5	IV	HSPCa	1861	T2cN0M1	GnRH agonists	FFPE sample from Biopsy	23/11/2016
13	84	4+5	III	HSPCa	80.4	T3aN0M0	GnRH antagonists	FFPE sample from Biopsy	01/06/2017
14	59	4+5	IV	HSPCa	54	T4N1M0	GnRH agonists	FFPE sample from Biopsy	16/04/2010
15	83	4+5	IV	HSPCa	261	T4N0M0	BXO	FFPE sample from Biopsy	14/08/2014
16	76	4+5	III	HSPCa	9.9	T3N0M0	BXO	FFPE sample from Biopsy	29/11/2011
17	81	4+5	III	HSPCa	145	T3N0M0	LHRH agonist	FFPE sample from Biopsy	06/04/2011
18	73	3+4	IV	HSPCa	494	T4N0M1	LHRH agonist	FFPE sample from Biopsy	12/08/2010
19	72	4+5	III	HSPCa	121	T3N0M0	BXO	FFPE sample from Biopsy	04/03/2010
20	66	5+5	IV	HSPCa	2647	T3N1M1	GnRH antagonists	FFPE sample from Biopsy	30/11/2016
21	81	5+5	IV	HSPCa	776	TxN0M1	BXO	FFPE sample from Biopsy	03/02/2016
22	67	5+5	IV	HSPCa	2105	T2cN0M1	BXO	FFPE sample from Biopsy	11/01/2017
23	81	5+5	IV	HSPCa	13.2	T3bN0M1	GnRH agonists	FFPE sample from Biopsy	15/02/2017
24	75	5+5	IV	HSPCa	625	T4N1M1	GnRH agonists	FFPE sample from Biopsy	12/07/2017

25	78	4+5	IV	HSPCa	214	T3bN1M1	GnRH antagonists	FFPE sample from Biopsy	16/08/2017
26	53	5+5	IV	HSPCa	73.4	T3N1M1	GnRH antagonists	FFPE sample from Biopsy	29/11/2017
27	76	5+4	IV	HSPCa	1334	T3bN1M1	GnRH antagonists	FFPE sample from Biopsy	09/11/2020
28	74	4+5	IV	HSPCa	195	T2cN1M0	GnRH antagonists	FFPE sample from Biopsy	23/11/2020
29	84	4+5	IV	HSPCa	250	T3bN1M1	LHRH agonist +Enzalutamid e	FFPE sample from Biopsy	30/12/2020
30	70	4+5	IV	HSPCa	2058	TxN0M1	GnRH antagonists	FFPE sample from Biopsy	30/12/2020
31	80	5+4	IV	CRPC	11	T4N0M1	Enzalutamide	FFPE sample from TURP removal	10/08/2020
32	78	4+5	IV	CRPC	2086	T2cN0M1	Enzalutamide	FFPE sample from TURP removal	06/11/2019
33	87	4+5	IV	CRPC	20.7	T4N0M0	Bicalutamide	FFPE sample from TURP removal	09/09/2019
34	68	4+5	IV	CRPC	226	T4N1M1	Chemotherapy	FFPE sample from TURP removal	19/09/2018
35	84	5+4	IV	CRPC	395	T4N0M1	Radiotherapy	FFPE sample from TURP removal	06/07/2015
36	80	5+4	IV	CRPC	16.5	T4N0M1	Bicalutamide	FFPE sample from TURP removal	03/03/2015
37	85	NA	IV	CRPC	11.3	T3N1M1	Bicalutamide	FFPE sample from TURP removal	14/01/2015
38	77	4+4	IV	CRPC	945	T4N0M1	Radiotherapy	FFPE sample from TURP removal	06/08/2014
39	76	5+4	IV	CRPC	150	T4N1M1	Abiraterone	FFPE sample from TURP removal	06/01/2014
40	88	5+4	IV	CRPC	557	T2cN1M1	Abiraterone	FFPE sample from TURP removal	06/07/2020
41	67	5+5	IV	CRPC	7.8	T3aN0M1	Bicalutamide	FFPE sample from TURP removal	22/06/2020
42	81	4+4	IV	CRPC	22.6	T4 N0M1	Abiraterone	FFPE sample from TURP removal	10/12/2018
43	89	5+5	IV	CRPC	223	TxN0M1	NA	FFPE sample from TURP removal	03/03/2017
44	85	5+4	IV	CRPC	830	TxN0M1	BXO	FFPE sample from TURP removal	20/07/2016

45	68	5+5	IV	CRPC	46.7	T4N1M1	Chemotherapy +Bicalutamide	FFPE sample from TURP removal	20/04/2016
46	68	5+4	IIB	CRPC	32.9	T2cN0M0	Bicalutamide	FFPE sample from TURP removal	23/12/2015
47	79	4+5	IV	CRPC	1771	TxNxM1	NA	FFPE sample from TURP removal	11/12/2018
48	75	4+5	IV	CRPC	205	T3N0M1	Abiraterone	FFPE sample from TURP removal	30/03/2021
49	66	5+4	IV	CRPC	307	T4NxM1	Abiraterone	FFPE sample from TURP removal	14/05/2018
50	80	NA	IV	CRPC	71	T2cN0M1	Abiraterone	FFPE sample from TURP removal	11/11/2016

For the screening cohort (small RNA sequencing), we used benign prostate tissues No.1-10, HSPCa tissues No.11-19, and CRPC tissues No.31-39 (No.11-19 and No.31-39 cases were paired HSPCa tissues and CRPC tissues); For the validation cohort, candidate piRNAs were validated in 10 benign prostate tissues (No. 1-10), 20 HSPCa tissues (No.11-30) and 20 CRPC tissues (No.31-50).

BXO: bilateral orchiectomy; FFPE: formalin-fixed paraffin-embedded; GnRH: Gonadotropin hormone-releasing hormone; LHRH: luteinizing hormone-releasing hormone; NA: not available, TURP: transurethral resection of the prostate.

Table S2: The sequences of piRNAs for Taqman RT-qPCR

piRNA name	Sequence 5'-3'
piR-2545834	TGGCAAGAAATGGGCTACATTTTCTCC
piR-908852	ATTTCTTCCCACCTCATGAGCTACA
piR-60094	AAACTGGGATTAGATACCCCACTGTGCTTAGCCC
piR-2651524	TAGGTCAAGGTGTAGCCCATAAGGT
piR-4447944	TAAGCTATCGGGCCCATACCCAGAAAATGTTGG
piR-4419185	TAGGGTTTACGATCTCGATGTTGGATCAGGAC
piR-2856682	CTGCTCACCAGTGGGCACTTGCAGGACGAGCACT
piR-597820	TGTATAAATAAGGATTATCCTGGGCATA
piR-1207232	AGGTCAAGGTGTAGCTCATGAGGTGGC
piR-2715339	TGACTAACGGAACAAGTTACCCTAGG

Table S3: Primers for mRNA RT-qPCR

Primers	Forward (5'-3')	Reverse (5'-3')
NEFH	GCACTGAAAAGCACCAAGGA	AGCCAATCCGACACTCTTCA
PLCD1	CCAGCCACTTAGCCACTACC	GAAGGCATAGTCCCGGATGG
PLIN2	GGCAGGCGACATCTACTCAG	GCACCTTGGTCCTGAGCATT
TENT5B	CCACTCCTTTCCCCCTTGTG	GGGTGGGAACGTTGACTTGA
U2AF1	TCTGTGACAACCTGGGAGAC	AGCTCTCTGGAAATGGGCTT
GJD3	CTTCCTACTTGCGGGCATTC	AACTCAGAAGGACAGGGTGG
AR	GGAGGCGACAGAGGGAAAAAG	CCTCCGAGTCTTTAGCAGCTT
KLK3/PSA	CAAGACTCAAGCCTCCCCAG	GCCAGTATTCCCCAGGACAC
GAPDH	CCCACTCCTCCACCTTTGAC	GGAGATTCAGTGTGGTGGGG

Table S4: The clean reads distribution of small RNA sequencing in each sample

Patient Number	All Qualified Reads	miRNA Mapping Reads	miRNA Mapping Rate (%)	piRNA Mapping Reads	piRNA Mapping Rate (%)	Others Reads	Others Mapping Rate (%)
Benign_1	27100318	22350392	82.473	362840	1.34	4387086	16.19
Benign_2	26233125	21814906	83.158	302788	1.15	4115431	15.69
Benign_3	24988367	20520209	82.119	274102	1.1	4194056	16.78
Benign_4	25890443	22267046	86.005	302520	1.17	3320877	12.83
Benign_5	25567067	21121192	82.611	403862	1.58	4042013	15.81
Benign_6	23903690	19824667	82.936	272302	1.14	3806721	15.93
Benign_7	25772632	21547749	83.607	239723	0.93	3985160	15.46
Benign_8	25810865	21466594	83.169	337179	1.31	4007092	15.52
Benign_9	25218962	20234420	80.235	339680	1.35	4644862	18.42
Benign_10	25263327	19044293	75.383	250967	0.99	5968067	23.62
HSPCa_11	23898003	13624792	57.012	414415	1.73	9858796	41.25
HSPCa_12	24350890	10098861	41.472	309057	1.27	13942972	57.26
HSPCa_13	24901521	12611650	50.646	375663	1.51	11914208	47.85
HSPCa_14	23881073	6024143	25.226	266673	1.12	17590257	73.66
HSPCa_15	23950552	8825219	36.848	391382	1.63	14733951	61.52
HSPCa_16	24018704	10139642	42.216	301381	1.25	13577681	56.53
HSPCa_17	23956058	8123676	33.911	290499	1.21	15541883	64.88
HSPCa_18	23853916	6160442	25.826	186047	0.78	17507427	73.39
HSPCa_19	23854805	9014382	37.789	288926	1.21	14551497	61.00

CRPC_31	24776830	9692976	39.121	228649	0.92	14855205	59.96
CRPC_32	24897893	13881942	55.755	326932	1.31	10689019	42.93
CRPC_33	23970405	16112234	67.217	394509	1.65	7463662	31.14
CRPC_34	23944764	8695987	36.317	218800	0.91	15029977	62.77
CRPC_35	24005558	9057754	37.732	249452	1.04	14698352	61.23
CRPC_36	24084154	7563198	31.403	223185	0.93	16297771	67.67
CRPC_37	22552518	7451607	33.041	399073	1.77	14701838	65.19
CRPC_38	24056624	7930027	32.964	263768	1.1	15862829	65.94
CRPC_39	23990386	7657374	31.919	217897	0.91	16115115	67.17

Table S5: The sequences of piRNA mimic/inhibitor for transfection

piRNA mimics	Sequence (5'-3')
piR-4447944 mimic	UAAGCUAUCGGGCCCAUACCCAGAAAAUGUUGG with monophosphate at the 5' ends and 2'-O-methylation at the 3'-ends
NC-mimic	UUUGUACUACACAAAAGUACUG with monophosphate at the 5' ends and 2'-O-methylation at the 3'-ends
piRNA inhibitors	Sequence (5'-3')
piR-4447944 inhibitor	CCAACAUUUUCUGGGUAUGGGCCCGAUAGCUUA with 2'-O-methylation modification
NC-inhibitor	UCACAACCUCCUAGAAAGAGUAGA with 2'-O-methylation modification

Table S6: Antibodies related to western blot and flow cytometry

Antibodies	Dilution and Source
FITC Annexin V	5ul/test; BD Biosciences # 556547
Propidium Iodide (50 µg/ml)	5ul/test; BD Biosciences #556547
PI/RNase Staining Buffer	500ul/test; BD Biosciences #550825
AR	1:1000 (WB), Abcam #ab108341
PSA	1:1000 (WB), Abcam #ab53774
NEFH	1:1000 (WB), Abcam #ab207176
cleaved Caspase-3	1:1000 (WB), CST #9664T
PIWIL2	1:1000 (WB), Abcam #ab36764
PIWIL4	1:500 (WB), Santa Cruz #sc-517215
Histone H3	1:1000 (WB), Abclonal #A2348
β-Actin	1:5000 (WB), CST #4970
GAPDH	1:5000 (WB), Abcam #ab9485

Anti-Rabbit IgG HRP-linked	1:10000 (WB), CST #7074
Anti-Mouse IgG HRP-linked	1:10000 (WB), CST #7076

Table S7: The top 10 significantly upregulated piRNAs in CRPC

piRNA Name	HSPCa group vs. Benign group			CRPC group vs. HSPCa group		
	log2FC	p-value	adjusted p-value	log2FC	p-value	adjusted p-value
piR-2545834	2.48	8.44E-03	1.04E-02	4.95	2.67E-07	1.88E-06
piR-908852	2.94	5.99E-04	1.02E-03	4.26	6.57E-07	3.97E-06
piR-60094	2.67	5.15E-04	8.98E-04	3.78	5.97E-08	5.22E-07
piR-2651524	1.93	1.39E-02	1.66E-02	3.60	1.49E-06	7.83E-06
piR-4447944	3.30	4.11E-05	1.13E-04	3.57	3.43E-08	3.24E-07
piR-4419185	2.22	1.10E-02	1.33E-02	3.55	8.19E-06	3.26E-05
piR-2856682	1.68	2.58E-02	2.96E-02	3.25	4.57E-05	1.34E-04
piR-597820	3.34	1.04E-04	2.43E-04	3.10	9.47E-08	7.80E-07
piR-1207232	4.59	8.80E-07	4.80E-06	3.10	9.18E-09	1.05E-07
piR-2715339	2.94	1.01E-03	1.59E-03	3.03	2.70E-07	1.89E-06
